# Genetics and phenotypic heterogeneity of Dent disease: the dark side of the moon

**DOI:** 10.1007/s00439-020-02219-2

**Published:** 2020-08-29

**Authors:** Lisa Gianesello, Dorella Del Prete, Franca Anglani, Lorenzo A. Calò

**Affiliations:** Nephrology, Dialysis and Transplantation Unit, Kidney Histomorphology and Molecular Biology Laboratory, Department of Medicine-DIMED, University of Padua, Via Giustiniani n° 2, 35128 Padua, Italy

## Abstract

Dent disease is a rare genetic proximal tubulopathy which is under-recognized. Its phenotypic heterogeneity has led to several different classifications of the same disorder, but it is now widely accepted that the triad of symptoms low-molecular-weight proteinuria, hypercalciuria and nephrocalcinosis/nephrolithiasis are pathognomonic of Dent disease. Although mutations on the *CLCN5* and *OCRL* genes are known to cause Dent disease, no such mutations are found in about 25–35% of cases, making diagnosis more challenging. This review outlines current knowledge regarding Dent disease from another perspective*.* Starting from the history of Dent disease, and reviewing the clinical details of patients with and without a genetic characterization, we discuss the phenotypic and genetic heterogeneity that typifies this disease. We focus particularly on all those confounding clinical signs and symptoms that can lead to a misdiagnosis. We also try to shed light on a concealed aspect of Dent disease. Although it is a proximal tubulopathy, its misdiagnosis may lead to patients undergoing kidney biopsy. In fact, some individuals with Dent disease have high-grade proteinuria, with or without hematuria, as in the clinical setting of glomerulopathy, or chronic kidney disease of uncertain origin. Although glomerular damage is frequently documented in Dent disease patients’ biopsies, there is currently no reliable evidence of renal biopsy being of either diagnostic or prognostic value. We review published histopathology reports of tubular and glomerular damage in these patients, and discuss current knowledge regarding the role of *CLCN5* and *OCRL* genes in glomerular function.

**Electronic supplementary material:**

The online version of this article (10.1007/s00439-020-02219-2) contains supplementary material, which is available to authorized users.

## Introduction

Dent disease is a group of X-linked recessive renal disorders characterized by an incomplete renal Fanconi syndrome. Dent disease may vary in clinical presentation with proteinuria alone or in combination with nephrocalcinosis/nephrolithiasis, with or without chronic kidney disease (CKD) (Dent and Friedman 1964; Lloyd et al. 1996; Thakker 2000). Like most genetic disorders, the onset is usually in childhood (Blanchard et al. 2016). Asymptomatic cases are sometimes diagnosed in adult age, however, usually due to the early development of an idiopathic CKD (at 30–50 years old) (Wrong et al. 1994; Lloyd et al. 1997; Zaniew et al. 2018).

The gene most often involved is *CLCN5* (responsible for about 65% of cases), mutations of which are responsible for Dent disease type 1 (DD1) (Jentsch et al. 1995; Thakker 1997; Waldegger and Jentsch 2000). Mutations in the *OCRL* gene cause Dent disease type 2 (DD2) (Hoopes et al. 2005), which is identified in about 10–15% of patients with Dent disease (Lieske et al. 1993). The remaining 25–35% of patients have a Dent disease phenotype but neither of these mutations, and are classified as cases of Dent disease type 3 (DD3) (Anglani et al. 2015).

While finding *CLCN5* and *OCRL* mutations unquestionably leads to a diagnosis of Dent disease, it sheds no light on the disease’s phenotypic heterogeneity. The Dent disease phenotype is defined by the presence of low-molecular-weight proteinuria (LMWP), hypercalciuria, and at least one other sign, which may be nephrocalcinosis, nephrolithiasis, hematuria, hypophosphatemia, or renal insufficiency (Lieske et al. 1993). That said, more and more reports are describing subjects carrying *CLCN5* or *OCRL* mutations who present an incomplete phenotype. This phenotypic diversity is the dark side of the moon in Dent disease, as it can lead to it going un- or misdiagnosed and inappropriately treated, and even to pointless renal biopsies.

Mutations in the *OCRL* gene cause both DD2 and Lowe syndrome (Lewis et al. 1993), and a genotype–phenotype correlation has been suggested (Shrimpton et al. 2009; Hichri et al. 2011). Such a correlation in DD1 has yet to be clarified, despite several experimental in vitro data on *CLCN5* mutations.

All this goes to show how hard it can be to distinguish Dent disease patients from those with other genetic or acquired forms of renal Fanconi syndrome. A phenocopy is defined as “a phenotypic trait or disease that resembles the trait expressed by a particular genotype, but in an individual who is not a carrier of that genotype” (NCI Dictionary of Genetics Terms 2012), and this concept applies very well to genetic forms of renal Fanconi syndrome (Solano et al. 2014).

In this review, we discuss the heterogeneity of Dent disease, starting from its first description and focusing on its diverse genetic and phenotypic features. We also underscore the complexity of DD1 and DD2 clinical phenotypes, in an effort to highlight those clinical manifestations that can easily lead to Dent disease being misdiagnosed.

## Brief history

In 1964, Dent and Friedman described two English males presenting with rickets associated with hypercalciuria and tubular proteinuria of unknown origin (Dent and Friedman 1964). The authors reported complete radiological healing of the rickets with no growth in stature. Since there was no family history, they excluded a hereditary origin of this disease (Dent and Friedman 1964).

Thirty years later, Dent disease was investigated more thoroughly. In 1991, Frymoyer et al. reported a large kindred study on 162 members of a family from northern New York that had an X-linked hereditary form of nephrolithiasis with renal failure (XRN, MIM #310468) (Frymoyer et al. 1991). The nine subjects affected had calcium nephrolithiasis, proteinuria, nephrocalcinosis, urinary concentration defects, anomalies in the renal excretion of calcium, phosphate, potassium and uric acid, and renal insufficiency with no bone disease or renal tubular acidosis (Frymoyer et al. 1991). The only other X-linked proximal tubulopathy to have been reported until then was Lowe syndrome (MIM #309000) (Lowe et al. 1952; Silver et al. 1987), but the absence of neurological or ophthalmological features prompted the authors to rule it out (Frymoyer et al. 1991). Around the same time, Furuse et al. also reported on six male patients from two different families who had tubular proteinuria, aminoaciduria and hypercalciuria; four of them also had phosphaturia; and two had glycosuria (Furuse et al. 1992). The authors were unable to establish whether this disease, termed idiopathic low-molecular-weight proteinuria (MIM #308990), was X-linked or autosomal dominant.

The term Dent disease (MIM #300009) was first introduced in 1993 to classify a form of renal Fanconi syndrome usually presenting with LMWP, hypercalciuria, nephrocalcinosis and nephrolithiasis. Less frequently, it might be associated with aminoaciduria, phosphaturia, kaliuresis, glycosuria, uricosuria, and impaired urinary acidification (Pook et al. 1993). Given the complex etiology of renal Fanconi syndrome (e.g. in association with inherited disorders, or with chronic hypocalcemia leading to hyperparathyroidism, or with renal poisoning due to heavy metals), Pook et al. investigated the genetic bases of Dent disease (Pook et al. 1993). They also included one patient initially described by Dent and Friedman, their case No. 1 (Dent and Friedman 1964) in their cohort of patients, and they identified the loci responsible for this disease in the Xp11.22 region. Linkage analysis with markers mapping in Xp11.22 led the authors to conclude, however, that Dent disease and XRN were caused by two different genes (Pook et al. 1993). Thakker et al. further investigated the previously described family from North America, and located the genetic cause of XRN in the Xp11.22 region (Scheinman et al. 1993). In the same year, an Italian group reported on a four-generation family with a new form of X-linked recessive hypophosphatemic rickets (XLRH, MIM #300554). They hypothesized that the genetic defect might be attributable to the human equivalent of the *Gyro* murine gene, or, anyway, to another gene in the Xp11.2 region (Bolino et al. 1993).

A year later, Wrong et al. examined 25 English patients, including the 2 subjects originally studied by Dent and Friedman (Wrong et al. 1994). Twenty-two of these cases were members of 5 different families. Analyzing these families expanded the spectrum of clinical features from those originally described by Dent and Friedman (Dent and Friedman 1964). Wrong et al. noted that rickets/osteomalacia were less common than LMWP, hypercalciuria or renal stones, and confirmed the X-linked inheritance of the disease (Wrong et al. 1994). They were the first to consider a defective renal tubular reabsorption as the main abnormality of Dent disease, rather than the destruction of tubular cells. At the same time, Fisher et al. finally cloned and characterized the *CLCN5* gene, proposing it as a likely culprit behind Dent disease (Fisher et al. 1994, 1995).

In 1995, Igarashi et al. voiced doubts on whether there was a difference between XRN and Dent disease (Igarashi et al. 1995), but it was only in 1996 that Lloyd et al. added another piece to the puzzle. They proposed *CLCN5* as the candidate gene, not only for Dent disease, but also for XRN and XLRH, shedding light on the marked phenotypic heterogeneity of Dent disease (Lloyd et al. 1996). The role of *CLCN5* in both Dent disease and XRN was confirmed in 1997 by Lloyd et al. and by Nakazato et al. (Lloyd et al. 1997; Nakazato et al. 1997).

Then, it took until 2004 for another milestone to be reached, regarding the genetic heterogeneity underlying Dent disease. Hoopes et al. described 13 unrelated males with a Dent disease phenotype (LMWP, hypercalciuria and at least one among nephrocalcinosis, nephrolithiasis, renal insufficiency, hypophosphatemia or hematuria) without any *CLCN5* gene mutations (Hoopes et al. 2004). The authors suggested that other gene(s), not necessarily located on the X chromosome, might be at the root of the problem. The following year, using linkage analysis, the same group identified the *OCRL* gene, located in the Xq27–Xq27.1 region, as responsible for the Dent disease phenotype in 5 of their 13 cases (Hoopes et al. 2005). These five patients did not have the key features of Lowe syndrome (cataracts or metabolic acidosis), but two of them had neurological abnormalities (Hoopes et al. 2005). The term Dent disease type 2 (MIM #300555) was introduced to distinguish cases with mutations in the *OCRL* gene from those with *CLCN5* mutations (Dent disease type 1).

**Fig. 1:**
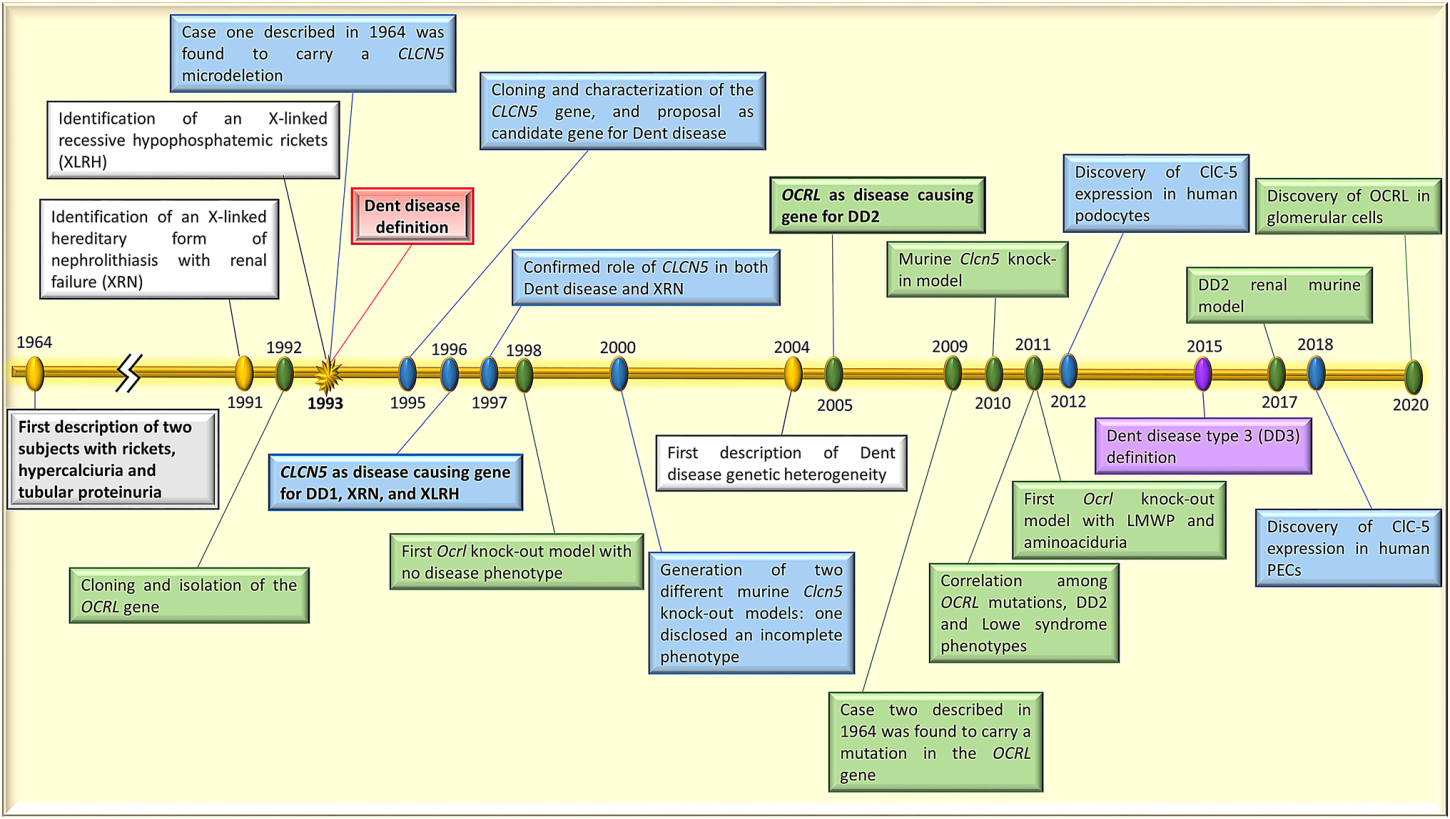
Time line showing the main steps in the history of Dent disease

A last element elucidating the two cases that Dent and Friedman first published (Dent and Friedman 1964) was added by an international collaborative study in 2009 (Shrimpton et al. 2009), which found that the genetic abnormality in case No. 2 was a mutation in exon 7 of the *OCRL* gene (p.(Met170Ilefs*1)). The diagnosis of Dent disease type 2 was supported by the patient’s mental retardation in addition to his renal symptoms (Dent and Friedman 1964). Unfortunately, this patient died before he knew his molecular diagnosis (Shrimpton et al. 2009). A summary of the key moments in the history of Dent disease is shown in Fig. 1.

## Genetic heterogeneity in Dent disease

Hoopes et al. first highlighted the genetic heterogeneity in Dent disease in the early 2000s. They described 32 unrelated males with a Dent disease phenotype, 19 (about 60%) of them with a mutated *CLCN5* gene (DD1), 5 (about 15%) with a mutated *OCRL* gene (DD2), and 8 (25%) with no mutations in either gene (DD3) (Hoopes et al. 2004, 2005). Several studies then tried to identify whether other genes were implicated in DD3 (Wu et al. 2009; Tosetto et al. 2009; Zhang et al. 2017a; Anglani et al. 2018; Gianesello et al. 2020a), but none have been identified to date.

It was the discovery of *CLCN5* and *OCRL* as disease-causing genes that helped to complete our understanding of the functional role of ClC-5 and OCRL proteins. In the next sections, we describe the in vitro and in vivo models used to study ClC-5 and OCRL function, and the pathogenic mechanisms behind Dent disease.

### The *CLCN5* gene and the ClC-5 protein

The first disease-causing gene associated with Dent disease was *CLCN5*. Initially named the human ClC-K2 protein coding gene, the *CLCN5* gene (MIM#300008, reference sequence NG_007159.2) was cloned 25 years ago (Fisher et al. 1994, 1995). Starting from a DD1 patient carrying a microdeletion in the Xp11.22 pericentromeric region (Pook et al. 1993), Fisher et al. identified a 170 kb gene encoding for a 2.2 kb transcript with a large 5′ untranslated region (UTR) (Fisher et al. 1995). The 5′UTR was predicted to include 2 strong promoters, and 1 weak one (Hayama et al. 2000; Tosetto et al. 2014), giving rise to 11 different mRNAs (Tosetto et al. 2014).

The *CLCN5* gene was subsequently located in the neighboring Xp11.23 chromosome band, and found to have 12 exons, with the ATG start site on exon 2 (Fisher et al. 1995). Five different *CLCN5* transcripts are produced, 2 of which (transcript variants 3 [NM_000084.5] and 4 [NM_001282163.1]) encode for the canonical 746 amino acid protein.

More than 250 different pathogenic variants of *CLCN5* have now been described (Mansour-Hendili et al. 2015; Gianesello et al. 2020b). Most of the reported mutations were missense (35%) or frameshift (31%), followed by nonsense (16%) and splicing mutations (10%), and large deletions (4%). With a lower frequency (1–2%), inframe deletions, complex mutations, Alu insertions and 5′UTR mutations have also been documented (Gianesello et al. 2020b).

The electrogenic chloride channel Cl^−^/H^+^ antiporter ClC-5 is the protein product of the *CLCN5* gene. Human ClC-5 was found to have a crucial role in the acidification of early endosomes of proximal tubular cells (Devuyst et al. 1999). Its renal expression is not limited to these cells, however. It has also been found in collecting duct α-intercalated cells, and in epithelial cells of the thick ascending limb of Henle’s loop (Devuyst et al. 1999). Proximal tubular cells also reportedly express ClC-5 in the brush border plasma membrane, where it is needed for low-molecular-weight (LMW) protein reabsorption (Günther et al. 1998; Devuyst et al. 1999).

The secondary structure of ClC-5 includes 18 α-helices (most of them transmembrane), a large intracytoplasmic C-terminus containing two cystathionine beta-synthase (CBS) domains, and an energy-sensing domain for the allosteric control mediated by ATP (Wu et al. 2003; Scott et al. 2004). Two glutamic acids are crucial to proper ClC-5 function: the “gating glutamate” Glu211 (Dutzler et al. 2003; Yin et al. 2004); and the “proton glutamate” Glu268 (Zdebik et al. 2008; Grieschat and Alekov 2012). ClC-5 possesses a unique *N*-glycosylation site at Asn408 (Wu et al. 2003).

### ClC-5 in vitro studies

Many in vitro studies have been performed to clarify the role of ClC-5 in Dent disease. Since ClC-5 was first thought to be a chloride channel, researchers often focused on the alteration of this property, particularly because it can be expressed at the plasma membrane. In 1996, 7 years before the ClC-5 protein structure as we know it today was first described (Wu et al. 2003), several groups began to investigate how *CLCN5* mutations alter the ClC-5 chloride current in *Xenopus laevis* oocytes (Lloyd et al. 1996). They found that missense or nonsense *CLCN5* mutations abolished or reduced this current (Lloyd et al. 1996). Friedrich et al. went on to study ClC-5 mutations in *Xenopus* oocytes and human embryonic kidney (HEK)-293 cells in parallel in an effort to establish whether the changes in chloride currents were mediated entirely by the altered ClC-5 or by a different channel that might be expressed in *Xenopus* but not in other species (Friedrich et al. 1999). Since no differences emerged between the two biological systems, the authors confirmed that the altered current depended entirely on ClC-5 (Friedrich et al. 1999). They also demonstrated that ClC-5 depends on pH, underscoring an important physiological role for this channel—particularly in view of its expression in the endocytotic pathway (Friedrich et al. 1999).

In 2005, Ludwig et al. examined the changes not only in the ClC-5 chloride current, but also in its surface expression (Ludwig et al. 2005). This study revealed that the previously identified reduced chloride current was not only due to a defective ClC-5 function alone, but also to a lower expression of the channel on the plasma membrane (Ludwig et al. 2005).

Other studies focused on the secondary and tertiary structure of the ClC-5 protein, looking at changes in CBS domain 2 (CBS2) (Carr et al. 2003), or the *N*-glycosylation site (Schmieder et al. 2007), more to see how the protein works than to clarify its role in Dent disease. Both studies reported a lower plasma membrane expression of ClC-5, but this was driven by different mechanisms. A defective CBS2 domain led to Golgi retention and a lack of ClC-5 in the endosomal vesicles and plasma membrane (Carr et al. 2003), and the absence of *N*-glycosylation caused a greater polyubiquitination, which contributed to an increased ClC-5 removal from the plasma membrane (Schmieder et al. 2007).

It was only in 2009 that some first attempts were made to classify the mutations by comparing the effects of *CLCN5* mutations with those on the *CFTR* gene (Smith et al. 2009; Grand et al. 2009). This classification is summarized in Table 1, and is applicable to missense, nonsense and frameshift mutations (Lourdel et al. 2012).

**Table 1:** Classification of Dent disease type 1 mutations

Class	Description
Class 1	Mutations that induce defective protein processing resulting in abolished electrical activity These mutations lead to ClC-5 retention within the endoplasmic reticulum, resulting in a defecting trafficking of the protein to the cell surface and/or to the early endosomes
Class 2	Mutations that induce a defect in protein processing and stability leading to a defective electrical activity These mutations lead to a defective ClC-5 that possesses a lower functionality and lower plasma membrane expression, but a normal distribution in the early endosomes
Class 3	Mutations that give no differences in the protein localization but induce a reduction in electrical activity These mutations lead to a properly folded proteins that can be correctly target to the plasma membrane and to early endosomes but display reduced or abolished chloride current

Several other researchers investigated how *CLCN5* mutations reflect on ClC-5 activity and/or plasma membrane expression (Yamamoto et al. 2000; Mo et al. 2004; Grand et al. 2011; Gorvin et al. 2013; D’Antonio et al. 2013; Satoh et al. 2016; Tang et al. 2016; Bignon et al. 2018; Chang et al. 2020), while others examined the changes due to mutations affecting *CLCN5* splice sites (Forino et al. 2004; Ramos-Trujillo et al. 2007; Inoue et al. 2020).

Although many mutations have been studied, a correlation between the type of mutation or the protein domain affected on one hand and the protein function/expression on the other has yet to be identified. Some studies on the same variant in different biological systems have even found opposite effects (Supplementary Table 1). All this research has produced evidence of a link between specific types of mutation and different cellular dysfunctions (Table 1), but these effects do not seem to correlate with the phenotypic heterogeneity seen in DD1 patients.

### In vivo models of Dent disease type 1

Two knock-out (KO) mouse models for *Clcn5* were generated in 2000 to shed light on the molecular mechanisms underlying Dent disease (Piwon et al. 2000; Wang et al. 2000). These models generated crucial insight on the mechanisms of proximal tubular dysfunction in DD1. They substantiated the involvement of the *Clcn5* gene in the pathogenesis of proximal tubular defects characterized mainly by LMWP and hyperphosphaturia. They also confirmed phenotypic heterogeneity as an important feature of this disease, as demonstrated by the lack of hypercalciuria and nephrolithiasis in one of the two KO models, despite both having the same genetic background (Piwon et al. 2000; Günther et al. 2003; Silva et al. 2003).

These in vivo models proved particularly useful for clarifying the altered mechanisms that lead to hypercalciuria. The findings indicated that, even in the absence of a decrease in 1,25-dihydroxyvitamin D3, hypercalciuria in *Clcn5* KO mice originates in the bone and kidney (Silva et al. 2003). It is not caused by intestinal calcium hyperabsorption, as previously supposed (Luyckx et al. 1999).

In 2010, to further explore the ClC-5 defects underlying DD1, Jentsch’s group created a knock-in (KI) mouse carrying a mutation in the “gating glutamate” Glu211 to convert ClC-5 from an Cl^−^/H^+^ exchanger into a Cl^−^ channel (Novarino et al. 2010). Intriguingly, when compared with the KO mice, these KI mice showed some similar traits (LMWP, hyperphosphaturia, hypercalciuria and impaired proximal tubular endocytosis), but differed in that their endosomal acidification was normal (Novarino et al. 2010). This goes to show the importance of Cl^−^ concentration in endosomal physiology—and in Dent disease.

In vivo studies also demonstrated the importance of ClC-5 in endocytosis and trafficking in proximal tubular cells, mainly by revealing the downregulation of megalin and cubilin at the brush border of KO mice (Christensen et al. 2003). Megalin and cubilin are both multiligand receptors with a key role in proximal tubular reabsorption. Their impaired expression could explain the greater loss of LMW proteins (including β_2_-microglobulin, transferrin, vitamin D, and retinol-binding proteins) in DD1 patients (Nielsen et al. 2016).

### The *OCRL* gene and the OCRL protein

The second disease-causing gene to be identified as responsible for Dent disease was the *OCRL* gene (MIM#300535, reference sequence NG_008638.1). This gene was first isolated in 1992 by Attree et al. starting from a patient with oculocerebrorenal syndrome of Lowe (OCRL)—hence the gene’s name—who carried an X-autosome translocation with the breakpoint at Xq25-q26 (Attree et al. 1992). The *OCRL* gene is located in the Xq26.1 region. It comprises 24 exons, with the ATG starting site in exon 2 (Attree et al. 1992; Nussbaum et al. 1997). Three transcripts have been described (transcript variant a [NM_000276.4], b [NM_001587.4] and c [NM_001318784.2]), which differ mainly in exon 18a, a 24 bp exon that may be either retained (isoforms a and c) or spliced out (isoform b) (Nussbaum et al. 1997). The three transcripts encode for three different proteins with 901 (isoform a), 893 (isoform b), and 902 (isoform c) amino acids, respectively. Isoforms a and b also differ in their tissue expression, the former being ubiquitous, the latter not expressed in the brain (De Matteis et al. 2017).

*OCRL* encodes for one member of the inositol polyphosphate-5-phosphatase enzyme family (OCRL), which is a shorter version of the inositol polyphosphate-5-phosphatase II expressed by platelets (Zhang et al. 1995). OCRL is primarily expressed in the *trans*-Golgi network, in early endosomes, and in lysosomes (Faucherre et al. 2003; Choudhury et al. 2005; Ghanekar and Lowe 2005; Oltrabella et al. 2015). It is widely expressed in the kidney, including the glomerulus and almost all of the tubular segments (Erb et al. 1997).

OCRL works as a phosphatase capable of removing the 5′ phosphate group from the phopshatidylinositol-4,5-bisphosphate (PIP_2_), a second messenger involved in vesicular trafficking (De Matteis et al. 2017). It consists of several protein domains: a 5-phosphatase catalytic domain, a pleckstrin homology (PH) domain (unable to bind phosphoinositides), an ASPM-SPD-2-Hydin (ASH) domain, and a RhoGAP-like domain with no GTPase-activating function, but capable of modulating how OCRL interacts with Cdc42 and Rac1 (Jefferson and Majerus 1995; Peck et al. 2002; Faucherre et al. 2003; Ponting 2006; Mao et al. 2009).

Studies in zebrafish showed that a lack of OCRL1 (the homolog of human OCRL) led to defects similar to those seen in cases of ClC-5 depletion, i.e. a defective tubular endocytosis and reduced megalin levels. Megalin accumulation in the endocytic compartments was also observed, supporting the role of OCRL in the recycling of this receptor too (Oltrabella et al. 2015).

Pathogenic variants of the *OCRL* gene were associated first with Lowe syndrome, and later with Dent disease (DD2) (Zhang et al. 1995; Hoopes et al. 2005). More than 140 *OCRL* mutations have been described so far (Ye et al. 2020), and identified throughout the gene. A few DD2 mutations are multi-exon deletions, while equal proportions of truncating (nonsense and frameshift) and non-truncating (missense) pathogenic variants account for the remainder (Lieske et al. 1993).

In 2011, a genotype–phenotype correlation was hypothesized, because nearly all mutations associated with DD2 are located among exons 1–7 that encompass the PH domain. Another reason is that most of the mutations causing Lowe syndrome are found among exons 8–23 that comprise the catalytic phosphatase, ASH, and RhoGAP-like domains (Hichri et al. 2011). A large international study on *OCRL* variants recently widened the range of exons leading to the DD2 phenotype by demonstrating that mutations related to Lowe syndrome can be located among exons 8 and 24, while exons 4–15 are involved in DD2 (Zaniew et al. 2018). Cases have also been reported in which *OCRL* mutations affecting exons at the 3′ side of exon 15 led to a DD2 phenotype (Sekine et al. 2014). Be that as it may, a relationship between genotype and phenotype has yet to be clearly established.

### OCRL in vitro studies

Compared with ClC-5, fewer studies have been conducted on the OCRL functional changes caused by the pathogenic variants identified in patients with Lowe syndrome or DD2. The first in vitro experiments were conducted in 1995 on fibroblasts and lymphoblastoid cells from patients with Lowe syndrome (Olivos-Glander et al. 1995; Suchy et al. 1995). The proximal tubular cells were investigated in 1998 (Zhang et al. 1998). It emerged that patients with Lowe syndrome lack OCRL expression in the Golgi and the lysosomes of all cell types analyzed (Olivos-Glander et al. 1995; Suchy et al. 1995; Zhang et al. 1998). Other studies found OCRL expression in the *trans*-Golgi network, due particularly to the enzyme’s ability to bind the Rac GTPase in both HEK-293-T and COS7 cells (Dressman et al. 2000; Faucherre et al. 2003).

In 2005, Choudhury et al. located OCRL in the endosomes of HeLa, normal rat kidney, and COS7 cells (Choudhury et al. 2005). Two separate groups also found an interaction between OCRL and clathrin-coated transport intermediates. These results point to the enzyme’s role in vesicular trafficking between the *trans*-Golgi and endosomes (Ungewickell et al. 2004; Choudhury et al. 2005). They also confirm a previous report from Hyvola et al. of OCRL targeting in both compartments mediated by Rab proteins (Hyvola et al. 2006).

OCRL involvement in the kidney was first investigated by Cui et al. (2010). These authors aimed to assess the effects of OCRL depletion on LMW protein uptake and megalin internalization kinetics in human (HK-2) and canine (MDCK) renal epithelial cells. They detected no effects (Cui et al. 2010), however, despite previous reports of a decreased megalin shedding in the urine of patients with Lowe syndrome (Norden et al. 2002). A study by Erdman et al. (2007) also located OCRL in early endosomes, shedding light on a possible involvement of OCRL in processes of receptor endocytosis and recycling by proximal tubular cells.

A step forward came with a study on proximal tubular cells from patients with Lowe syndrome, which identified an essential role for OCRL in early endosome function, and in vesicular trafficking defects due to a damaged F-actin filaments architecture (Vicinanza et al. 2011). The first change in actin filaments was found in fibroblasts from Lowe patients in 1995 (Suchy et al. 1995), a phenomenon later described in fibroblasts of DD2 patients too (Montjean et al. 2015). The mechanisms underlying this anomaly were further clarified by the discovery that OCRL interacts with cofilin, a protein implicated in F-actin remodeling (van Rahden et al. 2012), and the demonstration that the high curvature of the plasma membrane during endosome formation can act as a mechanical trigger for OCRL via the adaptor protein SNX9 (Daste et al. 2017).

Experiments conducted by Wu et al. in 2012 focused on the mechanisms underlying hypercalciuria (Wu et al. 2012). These authors described a dual role for OCRL in inhibiting TRPV6, a Ca^2+^ channel involved in calcium absorption in the intestinal epithelia. They found that: OCRL controls phosphatidylinositol 4,5-bisphosphate (PI(4,5)P_2_) levels in the cell membrane and, in turn, also TRPV6; and OCRL regulates TRPV6 trafficking via its Rab-binding domain. When OCRL is defective, there is a consequent loss of physiological TRPV6 inhibition, and this could pave the way to hypercalciuria through an increased intestinal absorption of Ca^2+^ (Wu et al. 2012).

### In vivo models of Dent disease type 2

In 1998, the first attempt to generate an *Ocrl* KO model gave rise to mice that developed none of the abnormalities seen in patients with DD2 or Lowe syndrome (Jänne et al. 1998). The authors surmised that loss of *Ocrl* in mice could be compensated by another protein, that they identified as Inpp5b, an enzyme with polyphosphate and PtInsP2 5-phosphatase activity (Jänne et al. 1998). Then *Inpp5b*^*−/−*^ mice also showed a near-normal phenotype. After crossing the *Ocrl*^*−/−*^ with the *Inpp5b*^*−/−*^ mice, there were no live-born embryos or littermates lacking in both enzymes, thus confirming the overlapping functions of the two enzymes, and the compensatory role of *Inpp5b* (Jänne et al. 1998).

Thirteen years later, the same group of researchers described a mouse model of DD2 and Lowe syndrome showing LMWP and generalized aminoaciduria, but no cataracts or growth defects (Bothwell et al. 2011). The Authors bypassed a previously-reported lethality problem in the double KO (*Ocrl*^*−/−*^ and *Inpp5b*^*−/−*^) mice by compensating the murine *Inpp5b* with the human paralog (*INPP5B*). The degree of this compensation was demonstrated to be dose-dependent. In fact, the mice homozygous for *INPP5B* had markedly more reduced LMWP and aminoaciduria than their heterozygous counterparts (Bothwell et al. 2011).

Festa et al. further investigated this model in 2019 (Festa et al. 2019). They examined male mice with and without *Ocrl* (*Ocrl*^*Y/*+^ and *Ocrl*^*Y/−*^, respectively) finding a proximal tubular dysfunction in the latter. They described this dysfunction as a partial renal Fanconi syndrome with albuminuria and LMWP but no polyuria, calciuria, glycosuria or phosphaturia associated with extrarenal symptoms (locomotor defects due to an impaired muscle apparatus). Behavior, learning and memory function, and vision were normal (Festa et al. 2019). On further analyzing the renal phenotype, the authors identified a defective receptor-mediated endocytosis that was probably due to a megalin misplacement; instead of localizing at the brush border of the proximal tubular cells, it was intracellular, particularly in the endosomes (Festa et al. 2019). Festa et al. also examined the cytological changes behind this phenomenon. They found that a stronger association between F-actin filaments and endosomes led to a trafficking defect that prevented megalin recycling to the plasma membrane (Festa et al. 2019).

In 2017, Inoue et al. used another biotechnological approach to study the DD2 renal phenotype, which involved a conditional *Inbb5b* gene deletion in the proximal tubular cells of global *Ocrl* KO mice (Inoue et al. 2017). These conditional double KO (cDKO) mice developed Fanconi syndrome with high urinary levels of vitamin D- and retinol-binding proteins, as well as albuminuria and phosphaturia. There was no difference in urinary calcium levels, compared with control mice (Inoue et al. 2017). The cDKO mice showed an impaired proximal tubular endocytosis, as seen previously in *Clcn5* KO mice (Piwon et al. 2000), supporting the hypothesis that both genes could be involved in a shared physiological process (Inoue et al. 2017).

Gliozzi et al. very recently published a study on zebrafish proximal tubular cells in which the *OCRL* gene was depleted by CRISPR/Cas9 genome editing (Gliozzi et al. 2020). They showed that OCRL has a role in cytokinesis. Its absence leads to an increase in multinucleated cells, and a shortening of megalin-expressing nephron segments, which should lie behind tubular proteinuria (Gliozzi et al. 2020). The authors proposed the dynamic remodeling of nephron segments as a new therapeutic target to slow the progression of kidney disease in patients with DD2 and Lowe syndrome (Gliozzi et al. 2020).

## Phenotypic heterogeneity of Dent disease

Since the clinical picture of Dent disease was first described (Pook et al. 1993), it has become widely accepted that the disease is best characterized by a triad of symptoms: LMWP, hypercalciuria, and nephrocalcinosis or nephrolithiasis (Pook et al. 1993). The marked heterogeneity of the disease’s clinical presentation nonetheless means that it is not unusual for cases to go unrecognized or be misdiagnosed.

Although Dent disease is a proximal tubulopathy, steroid resistant nephrotic syndrome (SRNS), focal segmental glomerulosclerosis (FSGS), or minimal change disease (MCD)—all typical consequences of glomerular dysfunction—are the clinical diagnosis most often advanced prior to the molecular diagnosis of Dent disease in childhood (Li et al. 2016; Trautmann et al. 2018). When Trautmann et al. performed genome-wide screening of a sample of SRNS patients, they found two brothers with DD1 who had initially been misclassified, because they had nephrotic-range proteinuria but no nephrocalcinosis or nephrolithiasis. It was only after genetic analyses that one of them revealed asymptomatic hypercalciuria (Trautmann et al. 2018).

In other reports, one case of DD1 and one of DD2 only showed persistent proteinuria (Salihu et al. 2018; Güngör et al. 2020), and the main clinical sign in a DD1 family was CKD of unknown etiology (Landini et al. 2020). Cases of brothers with the same *CLCN5* or *OCRL* mutations, but different phenotypes have also been described (Igarashi et al. 1998; Bökenkamp et al. 2009; Zhang et al. 2017a; Zaniew et al. 2017; Trautmann et al. 2018; Fischer et al. 2018). In the large kindred studied by Frymoyer et al. (1991), the only clinical manifestation in a 27-year-old male with a *CLCN5* mutation was hypercalciuria (Scheinman et al. 2000). Patients carrying the same pathogenic *OCRL* variant have been diagnosed sometimes as having Lowe syndrome, sometimes as cases of DD2 (Hichri et al. 2011).

Several authors conducted cohort studies and/or literature reviews in an effort to find features that DD1 and/or DD2 patients shared, and thus enable an earlier diagnosis and avoid unnecessary immunosuppressant therapies. Apart from LMWP, however, no other clinical sign was found common to patients with Dent disease (Hoopes et al. 2004; Tosetto et al. 2006; Bökenkamp et al. 2009; Sekine et al. 2014; Anglani et al. 2015; Mansour-Hendili et al. 2015; Blanchard et al. 2016; van Berkel et al. 2017; Zaniew et al. 2017, 2018; Ye et al. 2020).

DD1 patients have been described who carried pathogenic variants in both the *CLCN5* and other genes, such as the *NPHS2* gene (Zaniew et al. 2017) encoding for podocin (a key protein expressed by podocytes) or the *CFTR*, *SCNN1A* and *SCNN1B* genes (Zhang et al. 2017b), all of which are involved in cystic fibrosis (MIM #219700) or cystic fibrosis-like diseases. Two of three brothers described by Zaniew et al. had a mutation in the *NPHS2* gene as well as in the *CLCN5* gene, but they all three had different phenotypes (Zaniew et al. 2017). Two of these brothers (one with and one without the *NPHS2* mutation) underwent renal biopsy, which revealed different histological patterns. The ultrastructural findings were particularly intriguingly: the boy with the pathogenic variant of *NPHS2* had a normal podocyte ultrastructure, while his brother without the mutation had podocytes’ foot process effacement (Zaniew et al. 2017). Despite this, the boy with the double mutation rapidly progressed to end-stage renal disease (by 14 years of age).

On the other hand, the three males described by Zhang et al. (2017b) who carried additional mutations in the genes encoding for CFTR and the amiloride-sensitive or epithelial sodium channel (ENaC) had nothing in their clinical phenotype to induce physicians to rule out Dent disease. A couple of patients with cystic fibrosis reportedly had a Dent-like phenotype with significant LMWP and/or nephrotic-range proteinuria, and it was suggested that the ENaC cooperated with ClC-5 in proximal tubular endocytosis (Flores et al. 2003). This might explain the phenotypic overlap between these cases of cystic fibrosis and DD1.

Such reports go to show how complex the genetic background of individuals with Dent disease can be, and point to a possible role of modifier genes in defining the Dent disease phenotype.

### Clinical signs of Dent disease

Supporting the phenotypic similarities between DD1 and DD2 patients, the two cases initially reported by Dent and Friedman were subsequently identified as being caused by different mutations, affecting the *CLCN5* gene (in case No. 1) and the *OCRL* gene (in case No. 2) (Dent and Friedman 1964; Pook et al. 1993; Shrimpton et al. 2009).

To obtain a better picture of DD1 and DD2 patients, and seek clinical signs that might be distinctive of one rather than the other, we collected the clinical data published on males with DD1 (Hoopes et al. 2004; Tosetto et al. 2006; Bökenkamp et al. 2009; Sekine et al. 2014; Anglani et al. 2015; Mansour-Hendili et al. 2015; Blanchard et al. 2016; Zaniew et al. 2017; Ye et al. 2020; Sakakibara et al. 2020), and males with DD2 (Bökenkamp et al. 2009; Sekine et al. 2014; Zaniew et al. 2018; Ye et al. 2020; Sakakibara et al. 2020) (for details, see Supplementary Tables 2 and 3). Table 2 summarizes of the results of our analysis. Most studies describing Dent disease in the young are from Asia, but this is probably due to differences in health policy (for instance, Japan has an annual school screening program that leads to the early detection of proteinuria, and consequently of Dent disease (Sekine et al. 2014)).

**Table 2:** Clinical and biochemical signs observed in male subjects with *CLCN5* (DD1) or *OCRL* gene mutations (DD2)

Phenotype	DD1 (*n* = 772)	DD2^a^ (*n* = 143)	Chi-squared test (*p *value)
Age at diagnosis (years, range)	0.2–66	0.1–30.5	
Proteinuria	136/148 (92)	39/39 (100)	0.141
LMWP	719/720 (100)	134/134 (100)	1.000
Nephrotic range proteinuria	55/149 (37)	20/42 (48)	0.282
Hypercalciuria	556/686 (81)	104/122 (85)	0.323
Hematuria	88/145 (61)	16/32 (50)	0.361
Aminoaciduria	84/178 (47)	30/72 (42)	0.513
Hyperuricosuria	10/26 (38)	–	
Glycosuria	84/376 (22)	11/113 (10)	0.005
Hyperphosphaturia	62/228 (27)	9/46 (20)	0.372
Hypouricemia	25/62 (40)	3/5 (60)	0.699
Hypophosphatemia	78/240 (33)	5/49 (10)	0.003
Hypokalemia	80/257 (31)	6/56 (11)	0.003
Hypomagnesemia	7/36 (19)	1/19 (5)	0.309
Incomplete Fanconi syndrome	62/93 (67)	10/14 (71)	0.961
Complete Fanconi syndrome	8/154 (5)	1/38 (3)	0.810
Renal failure	159/565 (28)	39/89 (44)	0.004
Metabolic alkalosis	3/24 (13)	1/10 (10)	1.000
Metabolic acidosis	25/321 (8)	6/87 (7)	0.960
Nephrocalcinosis	366/664 (55)	32/127 (25)	0.000
Failure to thrive	33/122 (27)	27/50 (54)	0.001
Nephrolithiasis	95/388 (24)	9/66 (14)	0.000
Bone disorders	85/449 (19)	8/67 (12)	0.223
Neurological symptoms	–	4/16 (25)	
Intellectual disability	7/76 (9)	13/53 (25)	0.037
Cataract	1/9 (11)	8/87 (9)	1.000
Hypotonia	–	1/18 (5)	
Behavioral alterations	–	0/20(0)	

Data are shown as number of positive/total described cases (%). Pearson's Chi-squared test with Yates' continuity correction was used for the statistical analysis(R Core Team 2020). *p* < 0.05 was considered as significant

*LMWP* low-molecular-weight proteinuria, *DD1* Dent disease type 1, *DD2* Dent disease type 2

^a^Including one heterozygous female

Renal clinical signs of Dent disease mainly reflect the expression of both ClC-5 and OCRL in the proximal tubular epithelial cells (Christensen et al. 2003; Erdmann et al. 2007). In fact, the core feature of Dent disease is LMWP, found in almost all reported cases (Mansour-Hendili et al. 2015; Wang et al. 2016; Blanchard et al. 2016). Intriguingly, although LMWP is considered the clue to a diagnosis of Dent disease, two males with *CLCN5* mutations reportedly had no LMWP at the time of their diagnosis, but they did have other symptoms of Dent disease, such as hypercalciuria (Scheinman et al. 2000; Anglani et al. 2015). One, who belonged to a large family carrying the *CLCN5* mutation, subsequently developed LMWP (Anglani et al. 2015). We do not know whether this was also true of the other, a 27-year-old male from the family described by Frymoyer et al. (1991) (Scheinman et al. 2000). LMWP (as α_1_-microglobulin, β_2_-microglobulin, or retinol-binding protein) is not analyzed routinely, and this explains the discrepancy between the number of Dent cases reported in the literature and of the number of patients in which LMWP was tested. Proteinuria is commonly reported, sometimes even in the nephrotic range, reflecting a glomerular involvement—although Dent disease is a proximal tubulopathy.

One of the most common clinical signs in Dent disease types 1 and 2 is hypercalciuria, which was reported in more than 80% of patients with DD1 or DD2. This hypercalciuria was often described as intermittent, which could result in its underestimation.

Fanconi syndrome is the typical sign of a proximal tubular dysfunction. It is characterized by glycosuria, aminoaciduria, hyperphosphaturia and acidosis. Complete and incomplete Fanconi syndrome was reported equally often in cases of DD1 and DD2, usually in the incomplete form (in both types of Dent disease, it was incomplete in more than 60% of cases and complete in less than 10%). Glycosuria was found more frequently in DD1 than in DD2 patients (22% vs 10%, *p* < 0.01) (Table 2), while aminoaciduria and acidosis were equally common in both types. Although urinary glucose was not always measured (we collected data on 376/772 patients with DD1 and 113/142 with DD2), there was a significant difference between the two types of Dent disease. Sakakibara et al. (2020) also reported finding glycosuria more frequently in DD1 than in DD2, though the difference was not statically significant—probably due to the small number of cases considered. Supporting the involvement of ClC-5 in glucose handling by the proximal tubular cells (PTCs), Souza-Menezes et al. (2007) reported finding glycosuria in *Clcn5* KO mice. When the authors investigated the underlying mechanisms, they found a reduced GLUT2 expression in the PTCs, at both mRNA and protein levels, and lower serum insulin levels. Since megalin is important to the reabsorption of insulin by PTCs, and megalin expression was lower in *Clcn5* KO mice, the authors concluded that megalin downregulation could be one of the mechanisms behind the animals’ glycosuria (Souza-Menezes et al. 2007). A reduced megalin expression was also seen in *Ocrl* KO mice, so a similar defect in glucose maintenance might be suspected in DD2 as well, but no glycosuria was observed in these animals (Festa et al. 2019). The pathophysiological mechanisms underlying glycosuria in Dent disease have yet to be confirmed. It is hard to say whether these findings might be strong enough to discriminate DD1 from DD2, but glycosuria could point toward DD1 in the differential diagnosis.

Nephrocalcinosis and nephrolithiasis were detected more frequently in DD1 than in DD2 (*p* < 0.001 both) (Table 2). While a family history of kidney stones should also be considered in the differential diagnosis of Dent disease patients, there is also the possibility of de novo mutations (Cho et al. 2008), so patients with no such family history should not be excluded a priori. Uric acid is physiologically both excreted and reabsorbed by PTCs, and its levels are usually monitored in kidney stone patients. Males with DD1 reportedly had hypouricemia in 40% of cases, and hyperuricosuria in 38%, but only two studies reported data on uric acid in DD2, for three patients altogether (Cho et al. 2008; Blanchard et al. 2016).

Hematuria (usually microhematuria) is very common in patients with Dent disease, occurring in about a one in two cases of DD1 and DD2. It can be a sign not only of nephrolithiasis, but also of glomerulonephritis if associated with proteinuria. Due to their nephrotic-range proteinuria with or without LMWP, some patients with DD1 or DD2 reportedly underwent renal biopsy for suspected glomerulonephritis (as discussed later).

Bone abnormalities (rickets or osteomalacia) were reported in both DD1 and DD2 patients (in 19% and 13% of cases, respectively). These findings can be explained by two different mechanisms: (i) the previously described disruption of the calcium/phosphorus balance; and (ii) the downregulation of megalin and cubilin observed in DD1 and DD2 patients. Cubilin and megalin have a binding affinity for vitamin D-binding protein (Nielsen et al. 2016), and their impaired function leads to insufficient vitamin D reabsorption by the PTCs (Anglani et al. 2019).

In addition to the main clinical characteristics, there are also published reports on DD1 and DD2 patients with growth defects, including four cases with GH deficiency (Sheffer-Babila et al. 2008). Failure to thrive was described more frequently in DD2 than in DD1 (54% vs 27%, *p* < 0.01) and it is one of the few clinical signs that emerged as being significantly more associated with DD2 than with DD1 by our analysis (Table 2), and by Sakakibara et al. (2020). It is a typical feature of patients with *OCRL* mutations, who may have a very short stature, even below the 25th percentile (Utsch et al. 2006). This growth defect was also well documented in *Ocrl* KO mice (Bothwell et al. 2011).

Most males who develop Dent disease are diagnosed in childhood. When diagnosed in adulthood, this usually happens when they are referred to a nephrologist for idiopathic CKD. More than 30% of patients with DD1 or DD2 developed renal failure (Table 2), but this may be an underestimation, because most of the data collected on this aspect concerned cohorts of children, while we know that CKD is more likely to be seen in Dent disease patients from 30 to 50 years old (Lieske et al. 1993). End-stage renal disease (ESRD) was reported in 11% (12/81) of a sample of males with DD1 who were a mean 40 years old (Blanchard et al. 2016), but CKD (usually stage 2 or 3) could develop earlier on (from 10 to 30 years old). Renal function may reportedly decline at different rates among DD1 patients, even from the same family (Zaniew et al. 2017). Some males progressed rapidly to ESRD (as in a 14-year-old with DD1), while others might have CKD stage 2 or 3 for a lifetime (Zaniew et al. 2017). Sakakibara et al. reported a significantly worse renal function in patients with DD2 than in cases of DD1 (Sakakibara et al. 2020), a finding also supported by our analysis: renal failure occurred more in DD2 than DD1 (44% vs 28%, *p* < 0.01) (Table 2). We suggest that Dent disease should be suspected in young patients presenting with CKD of unknown origin with or without a family history of nephrolithiasis, and they should be tested for LMWP (if this has not been done already).

Regarding the mechanisms underlying CKD, Wang et al. showed that glomerular damage is common in Dent disease patients, and associated with a decline in kidney function (Wang et al. 2016). The authors also found an association between the presence of foot process effacement and a faster decline in kidney function, but no association with interstitial fibrosis, interstitial inflammation, tubular damage, or nephrocalcinosis (Wang et al. 2016). This is unexpected, because Dent disease is considered an inherited tubular disorder. Glomerular involvement in Dent disease is discussed in more detail later.

Dent disease type 2 reportedly differed from type 1 inasmuch as the former also involved extrarenal symptoms, and this led to DD2 being considered a mild variant of Lowe syndrome (Böckenhauer et al. 2012). But in the data, we collected on DD1 patients, we found that 1/9 patients had cataracts and 7/76 had intellectual disabilities (Table 2), revealing an unexpected dark side of the moon. Defects such as neurodevelopmental delay, cataract, and hypotonia are features of the oculocerebrorenal syndrome of Lowe (Hoopes et al. 2005), reflecting the broader expression of OCRL (kidney, eye, and brain) vis-à-vis ClC-5, which is mainly expressed in the kidney (Fisher et al. 1994; Olivos-Glander et al. 1995). These extrarenal clinical signs are to be expected, but are not always seen in patients with DD2 (Table 2). On the other hand, congenital cataract and intellectual disability were also described in children with DD1 in whom next-generation sequencing (NGS) studies revealed no *OCRL* mutations (Deng et al. 2020; Sakakibara et al. 2020). Dent disease patients are also usually referred to a nephrologist rather than a neurologist (unlike Lowe syndrome patients), so cognitive defects might go unreported, leading to an incomplete description of the clinical phenotype (Bökenkamp et al. 2009).

Park et al. suggested measuring creatine phosphokinase (CPK) and lactic dehydrogenase (LDH) to discriminate between DD1 and DD2 (Park et al. 2014). This is because children with DD2 often reportedly had high serum levels of these two muscle enzymes, while this was true of only a few children with DD1 (Utsch et al. 2006). Sakakibara et al. (2020) also noted significantly higher serum CPK and LDH levels in DD2 than in DD1 in their cohort of patients.

### Atypical phenotypes

As explained in previous paragraphs, DD1 is rarely associated with extrarenal symptoms, which are more common in DD2 (De Matteis et al. 2017), but these clinical signs may also override the classic Dent disease phenotype and prompt wrong diagnoses and treatments (Li et al. 2016). DD1 patients with extrarenal symptoms overlapping the DD2 phenotype have nonetheless been described (Table 2). As well as those listed in Table 2, other single case reports have been published: an individual with cataract (Langlois et al. 1998); another with attention deficit hyperactivity disorder (Sheffer-Babila et al. 2008); and one with mental retardation (Morimoto et al. 1998). These subjects were not tested for *OCRL* gene mutations, but one patient with co-inherited mutations in both the *CLCN5* and the *OCRL* genes had ocular anomalies, bone disease, and mild mental impairment (Addis et al. 2013).

DD1 has predominantly renal phenotype, because ClC-5 is expressed mainly in the kidney. There have been reports of DD1 patients with unusual symptoms too such as night blindness (Sethi et al. 2009; Becker-Cohen et al. 2012); growth hormone deficiency (Sheffer-Babila et al. 2008; Samardzic et al. 2011), and growth hormone deficiency complicated by Budd–Chiari syndrome (Platt et al. 2014). A patient initially diagnosed with Alport syndrome (due to hematuria, mild proteinuria, mild hearing loss, and diffuse thinning of the glomerular basement membrane) was subsequently classified as a case of DD1 using NGS (Yamamura et al. 2019).

Some specific comments are warranted on the description of cases with overlapping symptoms of Dent disease and Bartter-like syndrome, when a blended phenotype should be suspected due to the involvement of different segments of the nephron. Bartter syndrome (BS; MIM #s: type I, 601678; type II, 241200; type III, 607364; type IV, 602522; type IVb, 613090), and Gitelman syndrome (GS; MIM #263800) are autosomal recessive renal tubular disorders that usually present with hypokalemia, metabolic alkalosis, hyperreninemia, hyperplasia of the juxtaglomerular apparatus, and hyperaldosteronism. BS- and GS-causing genes encode proteins involved in renal electrolyte homeostasis that are known to be expressed in the thick ascending limbs of Henle’s loop, and in the distal convolute tubule (Blanchard et al. 2017). DD1 and BS may be diagnosed clinically in the same patient (Besbas et al. 2005; Bogdanović et al. 2010; Okamoto et al. 2012), but complete genetic screening was not undertaken, so we cannot know whether this blended phenotype is due to mutations in both the *CLCN5* and the BS- or GS-related genes or to an expansion of the DD1 phenotype. It has also been reported that Barttin, the protein altered in Bartter syndrome type 4, appears to regulate the subcellular localization and post-translational modification of ClC-5 (Wojciechowski et al. 2018). An abnormal interaction between these two proteins in the thick ascending limbs of Henle’s loop and the collecting duct might, therefore, lie behind the Bartter phenotype seen in some DD1 patients. Intriguingly, hypokalemia is a more frequent founding in DD1 (31% DD1 vs 11% DD2, *p* < 0.01) (Table 2).

Atypical phenotypes are more common in DD1 than in DD2; only a couple of patients have been described with a cutaneous disease (hidradenitis suppurativa) associated with a DD2 phenotype (Marzuillo et al. 2018, 2020).

### Dent disease type 3

It is becoming increasingly clear that *CLCN5* and *OCRL* gene mutations cannot account for all Dent disease patients. Some patients showing a Dent disease phenotype have no definite genetic diagnosis, and they are classified as cases of Dent disease type 3 (DD3) (Anglani et al. 2015).

Since 2005, when Hoopes et al. reported on *OCRL* has a second disease-causing gene (Hoopes et al. 2005), several cases of DD3 have been described (Hoopes et al. 2004; Utsch et al. 2006; Ramos-Trujillo et al. 2007; Sekine et al. 2007, 2014; Cho et al. 2008; Hichri et al. 2011; Anglani et al. 2015; Wang et al. 2016; Zhang et al. 2017b). The clinical features available for these patients are summarized in Table 3 (for more details, see Supplementary Table 4).

**Table 3:** Clinical and biochemical signs reported in male subjects without *CLCN5* and *OCRL* gene mutations (DD3)

Phenotype	DD3^a^ (*n* = 64)
Age (years, range)	2–58
LMWP	64/64 (100)
Hypercalciuria	44/59 (75)
Glycosuria	5/7 (71)
Aminoaciduria	3/5 (60)
Hyperphosphaturia	9/17 (53)
Hematuria	1/6 (17)
Hypophosphatemia	6/13 (46)
Renal failure	13/58 (22)
Complete Fanconi syndrome	0/14 (0)
Nephrocalcinosis	19/34 (56)
Nephrolithiasis	8/27 (30)
Bone disorders	10/54 (19)

Data are shown as number of positive/total described cases (%)

*LMWP* low-molecular-weight proteinuria

^a^Including one female

Unsurprisingly, most of these features are closely linked to the canonical Dent disease phenotype. LMWP, hypercalciuria, and at least one other of the usual symptoms (nephrocalcinosis, nephrolithiasis, hematuria, hypophosphatemia, hyperphosphaturia, bone disorders, or renal failure) are generally among the inclusion criteria for Dent disease studies (Table 3). Changes typical of renal Fanconi syndrome (including aminoaciduria and glycosuria) have also been examined in subjects with DD3 (Table 3). Only five males were evaluated for the presence of extrarenal symptoms typically found in DD2 or Lowe’s syndrome patients, and none of them showed cataract, hypotonia or neurodevelopmental delay (Sekine et al. 2007).

DD3 have been reported far less frequently (64 cases) than DD1 or DD2 (772 and 143 cases, respectively), resulting in a prevalence of DD3 of about 6.5%—much lower than the 25% previously reported (Mansour-Hendili et al. 2015). Negative data are liable to the so-called non-reporting bias, however, meaning that many cases certainly go unreported. In other words, a prevalence of 25–35% for DD3 is probably a more accurate estimation (Mansour-Hendili et al. 2015).

In the past, many authors tried to identify other disease-causing genes. *CLCN4*, the gene encoding for the ClC-4 channel, was the first to be investigated because of its location on the X chromosome and its close partnership with ClC-5 in endosomal acidification and trafficking by PTCs (Ludwig and Utsch 2004; Wu et al. 2009). No *CLCN4* mutations were found, however. Cofilin, a protein partner of both ClC-5 and OCRL in LMW protein endocytosis (Hryciw et al. 2003; van Rahden et al. 2012), was investigated too, again with no pathogenic variants coming to light (Wu et al. 2009). The same can be said of the sequencing of the *SLC9A6* gene encoding for the Na/H exchanger NHE6, and the *TMEM27* gene encoding for the amino acid transporter collectrin (Wu et al. 2009; Tosetto et al. 2009).

Some researchers focused on exploring genetic alterations in DD3 using more advanced technologies (Zhang et al. 2017b; Anglani et al. 2018; Gianesello et al. 2020a). NGS led to the discovery that the DD3 phenotype can be atypical or blended phenotypes of several known hereditary nephropathies. Zhang et al. (2017b) found a pathogenic variant in a DD3 patient’s *SCNN1A* gene, which encodes for the alpha subunit of the ENaC, emphasizing a resemblance between a couple of reported cases of cystic-fibrosis-like disease and Dent disease.

Another intriguing instance of blended phenotype was described in two DD3 patients first suspected of having DD1 or DD2, but high-throughput sequencing showed mutations in their *LRP2* gene, encoding for megalin, and this led to Donnai-Barrow syndrome clinical diagnosis (Anglani et al. 2018). Megalin and cubilin work together with ClC-5 and OCRL on the same endocytic pathway. In another subject with DD3, a possible digenic inheritance of two heterozygous mutations in the *LRP2* and *CUBN* genes, encoding for cubilin, was hypothesized (Gianesello et al. 2020a).

Whether mutations in a third (as yet unknown) gene could be responsible for DD3 remains to be seen, but previous reports do not support this hypothesis. NGS data endorse the theory that Dent disease type 3 is a set of atypical phenotypes of known hereditary nephropathies or blended phenotypes. Many genetic diseases can manifest with complete or incomplete Fanconi syndrome due to a proximal tubular dysfunction, and many clinical characteristics of different diseases can overlap. Full-blown classical phenotypes are rare. When multiple tubular defects coexist, they may conceivably give rise to a blended phenotype, and NGS might reveal multiple, interacting genes whose combined defects can explain individual phenotypes (Gianesello et al. 2020a).

## Glomerular damage in Dent disease

It is only in the last few years that glomerular involvement in Dent disease patients has begun to be considered, although the first evidence of advanced glomerulosclerosis in this setting was described in 1991 (Frymoyer et al. 1991). Glomerular disease was initially underestimated among the clinical signs of Dent disease, and/or considered merely as a consequence of the tubular damage. In the last decade, since the discovery of ClC-5 and OCRL expression in the glomerular compartment, a new theory has emerged according to which these two proteins’ loss of function leads to primary glomerular cell damage (Ceol et al. 2012; Gianesello et al. 2018; Preston et al. 2020). Glomerular damage was held responsible for the nephrotic-range proteinuria seen in more than 30% of Dent disease patients (Table 2). This led to podocytopathy being supposed in three brothers, and to podocyte-related genes (such as *NPHS2* and *WT1*) being investigated. Then, it took whole-exome screening for a pathogenic variant in the *CLCN5* gene to be identified (Zaniew et al. 2017).

Many studies describe DD1 and DD2 patients who underwent renal biopsy due to severe, even nephrotic-range proteinuria (Igarashi et al. 1998; Wang et al. 2016; Wong et al. 2017; Zaniew et al. 2017; Zhang et al. 2017b; Bao et al. 2019), which is the classic sign of potential glomerular damage. This means, on one hand, that several patients underwent renal biopsy unnecessarily. On the other, the specimens obtained further highlighted the heterogeneity of Dent disease, even in histopathological terms.

As summarized in Table 4, a variety of histopathological findings have been reported in DD1 and DD2 kidney biopsies (see Supplementary Table 5 for details). Patients may have normal glomerular and/or tubular compartments even on ultrastructural examination), or glomerulosclerosis (sometimes classified as FSGS, sometimes as focal global glomerulosclerosis—FGGS), with or without tubular atrophy or interstitial fibrosis.

**Table 4:** Histopathological signs observed in Dent disease kidney biopsies

	DD1	DD2
Glomerular histology		
Normal	12/76 (16)	4/17 (24)
Unspecified sclerosis	18/76 (24)	–
FGGS	16/76 (21)	1/17 (6)
FSGS	15/76 (20)	3/17 (18)
Mesangial proliferation	17/76 (22)	7/17 (41)
Minor glomerular abnormalities	7/76 (9)	4/17 (24)
Periglomerular fibrosis	5/76 (7)	–
Expansion of mesangial matrix	3/76 (4)	1/17 (6)
Immature glomeruli	2/76 (3)	–
Adherence to Bowman capsule	1/76 (1)	1/17 (6)
Other (perihyliar hyalinosis, ECM hyperplasia, collapsed tuft, podocytes' hypertrophy)	4/76 (5)	–
Tubular histology		
Normal	11/58 (19)	4/8 (50)
Tubular atrophy	17/58 (29)	1/8 (13)
Interstitial fibrosis	13/58 (22)	1/8 (13)
Calcification	10/58 (17)	–
Tubulointerstitial lesions	9/58 (16)	1/8 (13)
Calcium deposits	7/58 (12)	–
Intratubular proteinaceous casts	6/58 (10)	1/8 (13)
Interstitial inflammation	5/58 (9)	0/8 (0)
Nephrocalcinosis	4/58 (7)	
Vascular degeneration	3/58 (5)	–
Interstitial mononuclear cells infiltrate	2/58 (3)	–
Interstitial lymphocytes infiltrate	1/58 (2)	1/8 (13)
Acute tubular necrosis	–	1/8 (13)
Other (cortical fibrosis, interstitial chronic inflammation, chronic tubulointerstitial nephropathy with ischemic renal damage)	3/58 (5)	–
Immunofluorescence		
Negative	16/19 (84)	2/2 (100)
IgM deposits	3/19 (16)	–
C3 deposits	1/19 (5)	–
Transmission electron microscopy		
Normal	5/27 (19)	–
Foot process effacement	18/27 (67)	2/2 (100)
Electrondense deposits	1/27 (4)	–
Irregular GBM folding	–	1/2 (50)
Other (mesangial proliferation, collapsed glomeruli, GBM thickness, global sclerosis)	4/27 (15)	–

Data are shown as number of positive/total described cases (%)

*FSGS* focal segmental glomerulosclerosis, *FGGS* focal global glomerulosclerosis, *ECM* extracellular matrix, *GBM* glomerular basement membrane

There are patients (not necessarily relatives) with the same gene mutation and a different histology (Supplementary Table 5). No correlations have emerged between patients’ types of *CLCN5*/*OCRL* mutation and histopathological findings. This is hardly surprising, given the phenotypic heterogeneity of Dent disease, and the previously discussed sometimes incongruent results of in vitro experiments on *CLCN5* mutations (Supplementary Table 1).

Since glomerulosclerosis was a common finding in kidney biopsies from DD1 patients (Wang et al. 2016), a recent study by the Rare Kidney Stone Consortium (https://www.rarekidneystones.org) examined whether there were misdiagnosed cases of DD1 in two cohort of children with CKD or FSGS. No *CLCN5* mutations were identified among these patients, confirming the low rate of DD1 among pediatric populations with FSGS, as well as in other selected diagnoses that could be confused clinically with Dent disease (Beara-Lasic et al. 2020).

Glomerulosclerosis is a complex histological picture that may be due to podocyte injury and/or to parietal epithelial cell (PECs) activation (Fogo 2015). The first experimental evidence of a possible involvement of ClC-5 in the glomerular compartment was the discovery of its expression on human podocytes (Ceol et al. 2012), alongside its well-known protein partners megalin (Prabakaran et al. 2011) and cubilin (Prabakaran et al. 2012). Human podocytes were found capable of internalizing albumin, mainly through a cubilin–amnionless-mediated mechanism, and albumin overload was shown to induce an increase in ClC-5 expression in these cells (Gianesello et al. 2017). ClC-5, megalin and cubilin positivity was identified in human PECs too (Gianesello et al. 2018), expanding the spectrum of possible causes of glomerulosclerosis in patients with Dent disease. It was also shown that rat and murine PECs could internalize albumin under normal and overload conditions, in vivo and in vitro (Zhao et al. 2019), suggesting a role for ClC-5 in albumin uptake in these cells as well.

It was very recently reported that OCRL is more widely expressed in human glomeruli than ClC-5 (the former in podocytes, mesangial cells and endothelial cells, the latter in podocytes and PECs) (Preston et al. 2020). The authors suggested that OCRL is involved in regulating endocytic trafficking, actin cytoskeleton dynamics, and slit diaphragm maintenance, based on the close interaction between OCRL and CD2AP, a protein involved in slit diaphragm maintenance in podocytes (Preston et al. 2020). Mutations in the *OCRL* gene could disrupt these mechanisms, inducing glomerular damage as a result. The mesangial proliferation more frequently seen in DD2 than in DD1 (Table 4) could also be explained by the expression of OCRL, but not ClC-5, in mesangial cells. Experimental findings on the mechanisms leading to FSGS in patients with Dent disease have yet to be reported, so we can only suppose that they might be the same as those leading to nephrotic syndrome or FSGS in the general population—e.g. podocyte depletion, or podocyte—PEC contact (Fogo 2015). Wang et al. frequently found (albeit mild and segmental) podocyte foot process effacement in patients with Dent disease, and suggested that glomerulosclerosis in these patients might be the result of a combination of primary podocyte injury and a reaction secondary to tubulo-interstitial lesions (Wang et al. 2016). Given the interaction between ClC-5, OCRL, and cofilin, we speculate that the podocytes’ cytoskeletal structure might be disrupted, leading to cell detachment and glomerular damage. Further experimental data are needed to test this hypothesis, especially bearing in mind that the glomeruli of one *Clcn5* KO model revealed no histopathological damage (Cebotaru et al. 2005).

## Diagnosing Dent disease

Edvardsson et al. proposed a diagnostic algorithm for assessing potential cases of Dent disease in 2013 (Edvardsson et al. 2013). So did Zaniew et al. a few years later (Zaniew et al. 2017). In both models, the starting point for a diagnosis of Dent disease is the presence of proteinuria, subsequently identified as LMWP five times above the upper limit of normal, or at least markedly increased. The authors recommended screening for *CLCN5* and/or *OCRL* mutations in all males presenting with LMWP irrespective of any additional clinical features (nephrolithiasis or nephrocalcinosis, hypercalciuria, rickets, or CKD). Of course, any other identifiable cause of proximal tubular dysfunction should have already been excluded (Edvardsson et al. 2013; Zaniew et al. 2017).

Genetic screening of males with LMWP and at least one of the canonical associated clinical signs (hypercalciuria, nephrocalcinosis, nephrolithiasis, hematuria, and/or hypophosphatemia) resulted in a Dent disease detection rate that could vary from 66 to 92% (Hoopes et al. 2004, 2005; Ramos-Trujillo et al. 2007; Cho et al. 2008; Zhang et al. 2017b). On the other hand, Sekine et al. reported on the genetic screening of Japanese children using LMWP as the only inclusion criterion, and they reached a detection rate of 84% (Sekine et al. 2014). This latter finding supports the need to test for LMWP in males with proteinuria, and subsequently examining the *CLCN5* and/or *OCRL* genes in those patients with significantly elevated LMWP.

About 20–25% of cases of Dent disease are diagnosed as DD3, i.e. they are genetically unsolved (Lieske et al. 1993). As mentioned earlier, when NGS—either of target genes or using genetic panels—was used to investigate such cases, no other disease-causing genes were identified (Zhang et al. 2017b; Anglani et al. 2018; Gianesello et al. 2020a). Screening DD3 patients by NGS could nonetheless be a good way to identify hitherto unknown, atypical or blended phenotypes of other known hereditary nephropathies.

Reverse phenotyping could be described as an inverted diagnostic workflow that begins with genetic screening and ends with a clinical diagnosis. This approach is being used more and more frequently, particularly for those clinical cases not genetically solved by NGS. It helps clinicians to review a patient’s phenotype and arrive at the correct diagnosis. As mentioned earlier, reverse phenotyping helped to identify a couple of previously misdiagnosed Dent disease cases, expanding the spectrum of atypical Dent disease phenotypes (Trautmann et al. 2018; Yamamura et al. 2019). This procedure may generate more unexpected results in future.

Genetic testing is also useful for establishing healthy carrier status in mothers of males with Dent disease. In fact, males are more frequently affected than females, because it is an X-linked recessive disorder, and females may be heterozygous healthy carriers. Due to skewed X-inactivation, female carriers may also have some symptoms of the disease, such as LMWP or hypercalciuria (Reinhart et al. 1995). Nephrolithiasis, nephrocalcinosis, glycosuria, and aminoaciduria are reportedly rare in females (Wrong et al. 1994; Reinhart et al. 1995), although Li et al. (2016) found nephrolithiasis and nephrocalcinosis in heterozygous mothers more often than in their sons. ESRD has been described in only one female (Wrong et al. 1994).

Preimplant and prenatal genetic testing is not recommended for DD1, because the prognosis is good for most patients, and a clear correlation between genotype and phenotype is lacking (Devuyst and Thakker 2010).

## Therapy

As in many other genetic disorders, the focus of the clinical management of Dent disease patients is on the symptoms rather than on any specific therapy. The evidence to support most therapies in Dent disease is poor, which is reflected by highly variable treatment patterns in patients with *CLCN5* and *OCRL* mutations (Zaniew et al. 2017, 2018).

The main aim of pharmacological intervention is to reduce their proteinuria, hypercalciuria or bone disorders. Drugs are sometimes prescribed to modify calciuria and proteinuria under the assumption that these accelerate CKD progression. Moreover, recent data failed to show any association between nephrocalcinosis and progression of CKD in large cohorts of patients either with *CLCN5* or *OCRL* mutations (Blanchard et al. 2016; Zaniew et al. 2018).

Proteinuria is the key feature of the DD1 and DD2 phenotypes. Even though drugs inhibiting the renin–angiotensin–aldosterone system (RAAS) should not—in theory—be effective against tubular proteinuria, ACE inhibitors (ACEi) and angiotensin II receptor blockers (ARBs) proved effective in reducing proteinuria in Dent disease (though the data are inconclusive) (Blanchard et al. 2016; Zaniew et al. 2017). As suggested by Blanchard et al., the presence of histological glomerular damage and/or nephrotic-range proteinuria might at the base of ACEi/ARBs treatment (Blanchard et al. 2016). The reduction in albuminuria levels seen in some DD1 and DD2 patients may be due to the action of RAAS blockers on the proteinuria originating from glomerular damage. This is the picture that emerged in a study by Deng et al. on children with Dent disease, despite a decrease in albuminuria levels being reported in only half of those with confirmed glomerular disease (Deng et al. 2020). ACEi/ARBs are generally well tolerated by children with Dent disease. One patient reportedly developed hypotension, which was solved by administrating the drug in the morning (Deng et al. 2020). This retrospective study only examined the short-term effects (3 months), however; no studies on the long-term effects of ACEi/ARBs in DD1 and DD2 patients have been reported to date. To our knowledge, there are no studies which have addressed the effects of ACEi/ARBs on CKD progression in these patients. Moreover, clinical controversies on the treatment of patients with rare tubular/salt-losing disorders were risen by Kleta and Bokenhauer (2018), which underlined the risk of loss of volume homeostasis in subjects with Fanconi syndrome due to the impaired sodium reabsorption. In other words, the risk/benefit ratio must be well balanced when treating these patients with ACEi/ARBs.

So far, only two clinical trials on the treatment of Dent disease have been reported, neither of them specifically for DD2 or Lowe patients, and both focused on hypercalciuria, which is one of the most common clinical signs of DD1 and DD2 (Table 2).

Thiazide diuretics are commonly used in Dent disease to reduce hypercalciuria (Raja et al. 2002). The first clinical trial examined their use to prevent the nephrocalcinosis caused by the increased urinary calcium excretion in children with DD1 (ClinicalTrials.gov Identifier: NCT00638482). It reported a drop in urinary calcium levels, but with significant side effects, including hypokalemia in four and hypovolemia in all seven children enrolled. One boy developed symptomatic hypovolemia with cramps, and another had hyponatremia with severe extracellular dehydration that put a premature stop to the treatment. These findings led the authors to recommend caution when using diuretics in pediatric patients with Dent disease, and to suggest the use of hydrochlorothiazide only in recurrent stone formers (Lieske et al. 1993; Raja et al. 2002; Blanchard et al. 2008) as recommended for patients with nephrolithiasis (Gambaro et al. 2016). The data by Zaniew et al. (2018) also showed that *OCRL* mutated patients are more susceptible to severe dehydration, acute kidney injury and urinary tract infections. It clearly cautions against the use of thiazides and ACEi/ARBs in these children.

The second clinical trial (ClinicalTrials.gov Identifier: NCT02016235) aimed to establish whether phosphorus supplementation could reduce hypercalciuria in males with DD1 or DD2. The results of this study are still pending.

Nephrolithiasis is usually controlled with citrate supplementation (mainly potassium citrate). Citrates are also given to improve acidosis, and to slow the progression of CKD (Lieske et al. 1993; Zaniew et al. 2017), given the positive results obtained in *Clcn5* KO mice (Cebotaru et al. 2005). A citrate-rich diet in these mice was found to slow the loss of renal function, probably by reducing the production of TGFβ-1 by the tubular cells. This cytokine is known to contribute to kidney interstitial fibrosis and tubular atrophy, and the *Clcn5* KO mice fed a citrate-rich diet showed only minor histological damages (Cebotaru et al. 2005). No data on the long-term use of citrate in Dent disease are available as yet, however, therapy with citrates would be a good option, especially when hypocitraturia is present. Using this supplementation, one could expect to prevent stone formation, correct acidosis, improvement in bone density and height velocity, and most importantly slow CKD progression.

Another clinical feature of patients with Dent disease is bone involvement. Patients with high serum alkaline phosphatase levels responded well to vitamin D supplementation (Wrong et al. 1994). Here again, close follow-up of the treatment was recommended (monitoring calcemia, calciuria and 25-hydroxyvitamin D) to avoid exacerbating patient’s hypercalciuria and rising the risk of nephrocalcinosis (Blanchard et al. 2016).

A patient with DD1 and ESRD recently received a living kidney donation from his 75-year-old mother (Gambaro et al. 2019). This obligate carrier (with the p.(Arg467*) *CLCN5* mutation in heterozygosis) qualified for the donation, because she showed none of the clinical or biochemical signs of the Dent disease phenotype. This report points to new therapeutic options for patients with Dent disease, including intrafamilial kidney donation from obligate carriers after a careful assessment of their kidney function.

The therapeutic options described so far would need further investigation, especially considering the rarity of the disease and that most of the data available to us are obtained from retrospective studies. Moreover, we have to consider that we are facing with a fragile population in terms of age and clinical manifestations, in which the therapeutic options must be well-evaluated to avoid the already described undesirable side effects.

## Conclusions

Nowadays, there can be no doubt that the genetic and phenotypic heterogeneity of Dent disease prevent its prompt identification, and genetic screening is the only tool capable of establishing a diagnosis.

Dent disease should be considered in the differential diagnosis of male children presenting with glomerular signs (such as SRNS or idiopathic FSGS) or distal tubular signs (such as Bartter’s syndrome), or with idiopathic nephrolithiasis/nephrocalcinosis, or CKD. Physicians should then bear in mind, especially in this setting, that: “when you hear horseshoes, it’s usually a horse, but sometimes it’s a zebra”.

## Electronic supplementary material

Below is the link to the electronic supplementary material.

Supplementary file1 (DOCX 258 kb)

## Data Availability

All data analyzed for this study are included in this article (and the supplementary data files).

## Code availability

Statistical analyses were performed using R software version 3.4.1 (R Core Team 2020).
