# Supplementary material for: Genetics and phenotypic heterogeneity of Dent disease: the dark side of the moon
Source: Hum Genet. 2020 Aug 29;140(3):401–21. doi: 10.1007/s00439-020-02219-2 (PMC7889681; doi:10.1007/s00439-020-02219-2)
Supplement: Supplementary file 1 — Supplementary file1 (DOCX 258 kb) [file 439_2020_2219_MOESM1_ESM.docx]

Genetics and phenotypic heterogeneity of Dent disease: the dark side of the moon

Human Genetics

Lisa Gianesello, Dorella Del Prete, Franca Anglani*, Lorenzo A. Calò

*Nephrology, Dialysis and Transplantation Unit, Kidney Histomorphology and Molecular Biology Laboratory, Department of Medicine – DIMED, University of Padua, Padua, Italy*

*Corresponding author:

Dr. Franca Anglani

Nephrology, Dialysis and Transplantation Unit, Kidney Histomorphology and Molecular Biology Laboratory, Department of Medicine – DIMED, University of Padua, Via Giustiniani n° 2, 35128 Padova (Italy), phone: +390498212155, email: franca.anglani@unipd.it

**Supplementary Table 1:** Experimental *in vitro* data on ClC-5 mutant proteins

| **Type** | **Mutation** | **Chloride current** | **Plasma membrane expression** | **Other observations** | **Biological system** | **Ref** |
| --- | --- | --- | --- | --- | --- | --- |
| deletion | c.del132-241 |  |  | reduction in albumin and transferrin uptake, complete disruption of receptor-mediated endocytosis, defective endosomal acidification | Proximal epithelial tubular cells from urine of DD1 patients | (Gorvin et al. 2013) |
| frameshift | p.Trp173* |  | abolished | abolished protein expression | HEK293 | (Zhang et al. 2017) |
| frameshift | p.Ala232Glyfs*12 |  | abolished | Golgi retention | CHO-K1 and mIMCD-3 | (Carr et al. 2003) |
| frameshift | p.Ile243Asnfs*3 | abolished |  |  | Xenopus oocytes | (Yamamoto et al. 2000) |
| frameshift | p.Met517Ilefs*528 | abolished | abolished |  | Xenopus oocytes | (Ludwig et al. 2005) |
| insertion | 30:insH | reduced |  | reduction in albumin and transferrin uptake, blocking of megalin recycling, normal endosomal acidification | Xenopus oocytes, Proximal epithelial tubular cells from urine of DD1 patients | (Lloyd et al. 1997b; Gorvin et al. 2013) |
| missense | p.Trp22Gly | abolished | unaffected | disorders in endocytosis | Xenopus oocytes, LLC-PK1 and primary murine proximal tubular cells (wt and KO) | (Morimoto et al. 1998; Wang et al. 2005) |
| missense | p.Gly57Val | reduced | reduced | localized in late endosomes | Xenopus oocytes and HEK-MSR | (Lloyd et al. 1997a; Smith et al. 2009) |
| missense | p.Ser168Thr |  |  | reduced ion selectivity | Xenopus oocytes and HEK293 | (Friedrich et al. 1999) |
| missense | p.Gly179Asp | abolished | abolished | reduced protein expression, impaired *N*-glycosylation, ER retention | Xenopus oocytes and HEK293 | (Grand et al. 2009) |
| missense | p.Leu200Arg | abolished | abolished | reduced protein expression, impaired *N*-glycosylation, ER retention | Xenopus oocytes and HEK293 | (Lloyd et al. 1996; Grand et al. 2009) |
| missense | p.Ser203Leu | abolished | abolished | reduced protein expression, impaired *N*-glycosylation, ER retention | Xenopus oocytes and HEK293 | (Grand et al. 2009) |
| missense | p.Glu211Gly | reduced | unaffected | behavior as pure Cl- channel instead of Cl-/H+ antiporter, unaffected endosomal expression and acidification | Xenopus oocytes and HEK293T | (Bignon et al. 2018) |
| **Type** | **Mutation** | **Chloride current** | **Plasma membrane expression** | **Other observations** | **Biological system** | **Ref** |
| missense | p.Glu211Gln | unaffected | unaffected | behavior as pure Cl- channel instead of Cl-/H+ antiporter, defective endosomal acidification | Xenopus oocytes and HEK293 | (Satoh et al. 2016) |
| missense | p.Glu211Ala | unaffected | unaffected | drastic effect on gating and changed voltage dependence, abolished proton transport, abolished H+ transport and voltage dependence, normal endosomal acidification, behavior as pure Cl- channel instead of Cl-/H+ antiporter, defective endosomal acidification | Xenopus oocytes and HEK293 | (Friedrich et al. 1999; Picollo and Pusch 2005; Smith and Lippiat 2010; Satoh et al. 2016) |
| missense | p.Gly212Ala | reduced | unaffected | unaffected protein expression, *N*-glycosylation unaffected | Xenopus oocytes and HEK293 | (Grand et al. 2009) |
| missense | p.Cys219Arg | abolished | abolished | reduced protein expression, impaired *N*-glycosylation, ER retention | Xenopus oocytes and HEK293 | (Grand et al. 2009) |
| missense | p.Cys221Arg | abolished | abolished | reduced protein expression, impaired *N*-glycosylation, ER retention, misfolded in the ER, polyubiquinated and degraded by proteasome | Xenopus oocytes, HEK293, OK cells | (Ludwig et al. 2005; Grand et al. 2009; D’Antonio et al. 2013) |
| missense | p.Leu225Pro | abolished | abolished | altered N-glycosylation, ER retention | Xenopus oocytes and HEK293 | (Grand et al. 2011) |
| missense | p.Ser244Leu | reduced/ abolished | unaffected | altered Cl-/H+ stoichiometry | Xenopus oocytes, HEK293 and renal cortical tubular epithelial cells | (Lloyd et al. 1996; Grand et al. 2011; Tang et al. 2016; Chang et al. 2020) |
| missense | p.Ser244Ala | abolished |  | no changes in ion selectivity or in steady-state rectification | HEK293 and Xenopus oocytes | (Lloyd et al. 1996; Friedrich et al. 1999) |
| missense | p.Ser244Thr | reduced |  | no changes in ion selectivity or in steady-state rectification | HEK293 and Xenopus oocytes | (Friedrich et al. 1999) |
| missense | p.Gly260Val | abolished | reduced |  | Xenopus oocytes and HEK293 | (Grand et al. 2011) |
| missense | p.Glu268Ala | abolished |  | normal endosomal acidification | HEK293 | (Smith and Lippiat 2010) |
| missense | p.Ser270Arg | reduced/ abolished | absent | ER localization | Xenopus oocytes and HEK-MSR | (Igarashi et al. 1998; Smith et al. 2009) |
| missense | p.Tyr272Cys | abolished | unaffected |  | Xenopus oocytes and HEK293 | (Grand et al. 2011) |
| **Type** | **Mutation** | **Chloride current** | **Plasma membrane expression** | **Other observations** | **Biological system** | **Ref** |
| missense | p.Leu278Phe | reduced | reduced |  | Xenopus oocytes and HEK293 | (Igarashi et al. 1998; Grand et al. 2011) |
| missense | p.Arg280Pro | reduced | reduced | localized in early endosomes | Xenopus oocytes and HEK-MSR | (Lloyd et al. 1997b; Smith et al. 2009) |
| missense | p.Leu324Arg | abolished | abolished |  | Xenopus oocytes | (Ludwig et al. 2005) |
| missense | p.Gly333Arg | abolished |  | lower expression, impaired *N*-glycosylation | Xenopus oocytes and CHO | (Tanuma et al. 2007) |
| missense | p.Asn340Lys | abolished | abolished | altered N-glycosylation, ER retention | Xenopus oocytes and HEK293 | (Grand et al. 2011) |
| missense | p.Arg345Trp | reduced | reduced | reduced endosomal expression, ER localization, altered Cl-/H+ stoichiometry | Xenopus oocytes, HEK293 and renal cortical tubular epithelial cells | (Tang et al. 2016; Chang et al. 2020) |
| missense | p.Asn408Gln | reduced | unaffected | impaired *N*-glycosylation, increased polyubiquitination | Xenopus oocytes | (Schmieder et al. 2007) |
| missense | p.Gly462Val | abolished | abolished |  | Xenopus oocytes | (Ludwig et al. 2005) |
| missense | p.Leu469Pro | abolished | abolished | reduced protein expression, impaired *N*-glycosylation, ER retention | Xenopus oocytes and HEK293 | (Grand et al. 2009) |
| missense | p.Gly506Glu | abolished | abolished |  | Xenopus oocytes | (Lloyd et al. 1996; Ludwig et al. 2005) |
| missense | p.Gly512Arg | abolished |  |  | Xenopus oocytes | (Lloyd et al. 1997a) |
| missense | p.Gly513Glu | reduced/ abolished | abolished | altered *N*-glycosylation, ER retention | HEK-MSR, Xenopus oocytes and HEK293 | (Smith et al. 2009; Grand et al. 2011) |
| missense | p.Arg516Trp | reduced | unaffected/ abolished | ER localization | Xenopus oocytes and HEK-MSR | (Ludwig et al. 2005; Smith et al. 2009) |
| missense | p.Ser520Pro | reduced | unaffected | disorders in endocytosis, not efficiently targeted to endosomes | Xenopus oocytes, LLC-PK1 and primary murine proximal tubular cells (wt and KO) | (Lloyd et al. 1996; Wang et al. 2005) |
| missense | p.Ser520Ala | none |  |  | Xenopus oocytes | (Lloyd et al. 1996) |
| missense | p.Ser520Thr | none |  |  | Xenopus oocytes | (Lloyd et al. 1996) |
| missense | p.Leu521Arg | abolished | abolished |  | Xenopus oocytes | (Ludwig et al. 2005) |
| **Type** | **Mutation** | **Chloride current** | **Plasma membrane expression** | **Other observations** | **Biological system** | **Ref** |
| missense | p.Ile524Lys | reduced | abolished | ER localization | HEK-MSR | (Smith et al. 2009) |
| missense | p.Glu527Asp | abolished/ reduced | reduced | localized in both early and late endosomes | Xenopus oocytes and HEK-MSR | (Lloyd et al. 1997a; Smith et al. 2009) |
| missense | p.Ser540Ala | reduced |  | no changes in ion selectivity or in steady-state rectification | HEK293 and Xenopus oocytes | (Friedrich et al. 1999) |
| missense | p.Ser540Thr | reduced |  | no changes in ion selectivity or in steady-state rectification | HEK293 and Xenopus oocytes | (Friedrich et al. 1999) |
| missense | p.Lys546Glu | abolished | reduced |  | Xenopus oocytes and HEK293 | (Grand et al. 2011) |
| missense | p.Trp547Gly | reduced | reduced |  | Xenopus oocytes and HEK293 | (Grand et al. 2011) |
| missense | p.Asn565Lys | reduced |  | no changes in rectification but influence pore properties | HEK293 and Xenopus oocytes | (Friedrich et al. 1999) |
| missense | p.Gly600Val |  | reduced | reduced core glycosylation, localized in early endosomes | HEK293 | (Zhang et al. 2017) |
| missense | p.Asp601Val | unaffected |  |  | Xenopus oocytes | (Yamamoto et al. 2000) |
| missense | p.Thr657Ser | unaffected | unaffected | unaffected Cl-/H+ stoichiometry | Xenopus oocytes, HEK293, and renal cortical tubular epithelial cells | (Tang et al. 2016; Chang et al. 2020) |
| nonsense | p.Trp279* | abolished |  |  | Xenopus oocytes | (Lloyd et al. 1996) |
| nonsense | p.Arg347* | abolished | reduced/ abolished |  | Xenopus oocytes and COS-7 | (Morimoto et al. 1998; Mo et al. 2004; Ludwig et al. 2005) |
| nonsense | p.Tyr549* |  | abolished | ER localization | HEK293 | (Zhang et al. 2017) |
| nonsense | p.Tyr617* | abolished | reduced |  | Xenopus oocytes and COS-7 | (Mo et al. 2004) |
| nonsense | p.Gln629* | abolished | abolished | altered Cl-/H+ stoichiometry, ER and Golgi localization, no expression in early endosomes | Xenopus oocytes, HEK293 and renal cortical tubular epithelial cells | (Tang et al. 2016; Chang et al. 2020) |
| **Type** | **Mutation** | **Chloride current** | **Plasma membrane expression** | **Other observations** | **Biological system** | **Ref** |
| nonsense | p.Arg637* |  |  | reduction in albumin and transferrin uptake, blocking of megalin recycling, defective endosomal acidification | Proximal epithelial tubular cells from urine of DD1 patients | (Gorvin et al. 2013) |
| nonsense | p.Arg648* | reduced/ abolished | reduced/ abolished | Golgi retention | Xenopus oocytes, COS-7, CHO-K1 and mIMCD-3 | (Lloyd et al. 1996, 1997a; Igarashi et al. 1998; Carr et al. 2003; Mo et al. 2004; Ludwig et al. 2005) |
| nonsense | p.Val655* | abolished | reduced |  | Xenopus oocytes and COS-7 | (Mo et al. 2004) |
| nonsense | p.Arg704* | abolished | unaffected/ reduced/ abolished | Golgi retention, disorders in endocytosis, not efficiently targeted to endosomes | Xenopus oocytes, COS-7, CHO-K1, mIMCD-3, LLC-PK1 and primary murine proximal tubular cells (wt and KO) | (Lloyd et al. 1996; Igarashi et al. 1998; Carr et al. 2003; Mo et al. 2004; Wang et al. 2005) |
| nonsense | p.Arg718* | abolished | abolished | Reduced protein expression, impaired *N*-glycosylation, ER retention, misfolded in the ER, polyubiquinated and degraded by proteasome | Xenopus oocytes, HEK293 and OK cells | (Grand et al. 2009; D’Antonio et al. 2013) |
| splicing | c.1348-1G>A |  |  | First 26 bases of exon 9 were spliced out resulting in the loss of the CBS domain, impaired endosomal acidification, downregulation of megalin | Proximal tubule cells from the patient's urine | (Matsumoto et al. 2018) |

**Supplementary Table 2**: Clinical and biochemical data collected for DD1 patients

| **Clinical sign** | **TOT** | (Akuta et al. 1997) | (Nakazato et al. 1997) | (Akuta et al. 1997) | (Hoopes et al. 1998) | (Igarashi et al. 1998) | (Kelleher et al. 1998) | (Langlois et al. 1998) | (Morimoto et al. 1998) | (Nakazato et al. 1999) | (Cox et al. 1999) |
| --- | --- | --- | --- | --- | --- | --- | --- | --- | --- | --- | --- |
| **n** | 772 | 12* | 2* | 6* | 10* | 8* | 5* | 2* | 5* | 3* | 8* |
| **Population** |  | Japanese | Japanese | Japanese | American | Japanese | American | Canadian | Japanese | Japanese | European |
| **Age (years, range)** | 0.2-66 | 7-20 | 7-7 | 3-14 | 2-54 | 5-45 | 2-22 | 1.5-6.5 | 3-23 | 5-6 | 2-36 |
| **Proteinuria** | 136/148 | 11/12 | 2/2 | 6/6 | 7/10 | 7/8 | 5/5 | 2/2 | 5/5 | 3/3 | - |
| **Nephrotic range proteinuria** | 55/149 | 1/12 | 1/2 | 0/6 | - | 0/8 | 2/5 | 0/2 | 1/5 | 3/3 | - |
| **LMWP** | 719/720 | * | * | * | * | * | * | * | * | * | * |
| **Hypercalciuria** | 556/686 | * | * | * | * | * | * | * | * | * | * |
| **Hyperphosphaturia** | 62/228 | * | * | * | * | * | * | * | * | * | * |
| **Aminoaciduria** | 84/178 | * | * | * | * | * | * | * | * | * | * |
| **Glycosuria** | 84/376 | * | * | * | * | * | * | * | * | * | * |
| **Nephrocalcinosis** | 366/664 | * | * | * | * | * | * | * | * | * | * |
| **Renal failure** | 159/565 | * | * | * | * | * | * | * | * | * | * |
| **Hypokalemia** | 80/257 | * | * | * | * | * | * | * | * | * | * |
| **Fanconi incomplete** | 62/93 |  |  |  |  |  |  |  |  |  |  |
| **Fanconi complete** | 8/154 |  |  |  |  |  |  |  |  |  |  |
| **Hyperuricosuria** | 10/26 |  |  |  | 1/1 |  |  |  |  |  |  |
| **Hematuria** | 88/145 | 9/12 |  |  | 7/10 | 7/8 |  | 2/2 | 3/5 | 3/3 |  |
| **Hypophosphatemia** | 78/240 | 0/12 |  |  | 0/2 | 1/8 | 0/5 |  | 1/5 |  |  |
| **Hypocalcemia** | 4/78 | 0/12 |  | 0/6 | 0/2 | 1/8 | 0/5 | 0/1 | 0/5 |  |  |
| **Hypomagnesemia** | 7/36 |  |  | 0/6 |  |  |  |  |  |  |  |
| **Failure to thrive** | 33/122 |  |  |  | 0/1 |  |  |  | 1/1 |  |  |
| **Nephrolithiasis** | 95/388 |  |  |  | 5/10 |  | 4/5 |  |  |  |  |
| **Hyponatremia** | 3/17 |  |  |  |  |  |  |  |  |  |  |
| **Hypochloremia** | 4/37 | 0/12 |  |  |  | 1/8 |  |  |  |  |  |
| **Hypouricemia** | 25/62 |  |  |  | 1/1 |  |  |  |  |  |  |
| **Metabolic alkalosis** | 3/24 |  |  |  |  |  | 0/5 |  |  |  |  |
| **Metabolic acidosis** | 25/321 |  |  |  |  |  |  |  |  |  |  |
| **Bone disorders** | 89/469 |  | 0/2 |  | 2/3 |  | 0/5 | 1/2 | 1/1 | 0/3 | 3/8 |
| **Hypertension** | 0/7 |  |  |  |  |  |  |  |  |  |  |
| **Intellectual disability** | 7/76 |  |  |  |  |  |  |  |  |  |  |
| **Cataract** | 1/9 |  |  |  |  |  |  |  |  |  |  |

LMWP: Low-molecular-weight proteinuria; *already reported in Bökenkamp

| **Clinical sign** | (Igarashi et al. 2000) | (Yamamoto et al. 2000) | (Takemura et al. 2001) | (Carballo-Trujillo et al. 2003) | (Matsuyama et al. 2004) | (Hoopes et al. 2004) | (Cheong et al. 2005) | (Tosetto et al. 2006) | (Ramos-Trujillo et al. 2007a) | (Copelovitch et al. 2007) | (Ramos-Trujillo et al. 2007b) |
| --- | --- | --- | --- | --- | --- | --- | --- | --- | --- | --- | --- |
| **n** | 3* | 6* | 2* | 5* | 7* | 19* | 3* | 21* | 4 | 2 | 7* |
| **Population** | Japanese | Global | Japanese | Spanish | Japanese | Global | Korean | Italian | Spanish | American | Spanish |
| **Age (years, range)** | 3-29 | 1-43 | 13-16 | 0.7-10 | 1-12 | 2.5-39 | 0.2-9 | 5-29 | 2-14 | 9-12 | - |
| **Proteinuria** | 3/3 |  | 2/2 | 5/5 | 6/7 | - | 3/3 | 6/6 | 4/4 | 2/2 | - |
| **Nephrotic range proteinuria** | 0/3 |  | 0/2 | 1/5 | 0/7 | - | 1/3 | 4/6 | 1/4 |  | - |
| **LMWP** | * | * | * | * | * | * | * | * | 4/4 | 2/2 | * |
| **Hypercalciuria** | * | * | * | * | * | * | * | * | 4/4 | 2/2 | * |
| **Hyperphosphaturia** | * | * | * | * | * | * | * | * |  |  | * |
| **Aminoaciduria** | * | * | * | * | * | * | * | * |  | 1/1 | * |
| **Glycosuria** | * | * | * | * | * | * | * | * |  | 0/1 | * |
| **Nephrocalcinosis** | * | * | * | * | * | * | * | * | 2/4 |  | * |
| **Renal failure** | * | * | * | * | * | * | * | * | 1/4 |  | * |
| **Hypokalemia** | * | * | * | * | * | * | * | * |  | 0/2 | * |
| **Fanconi incomplete** |  |  |  |  |  |  |  |  |  |  |  |
| **Fanconi complete** |  |  |  |  |  |  |  |  |  |  |  |
| **Hyperuricosuria** |  |  | 0/2 |  |  |  | 1/3 | 3/9 |  |  |  |
| **Hematuria** | 2/3 |  | 0/2 | 5/5 | 5/7 | 16/19 | 1/3 |  |  | 2/2 |  |
| **Hypophosphatemia** | 0/2 |  | 0/2 | 2/5 | 1/6 | 9/18 | 0/3 | 3/20 |  | 0/2 |  |
| **Hypocalcemia** | 0/2 |  | 0/2 | 0/5 | 0/6 |  | 0/3 | 0 |  | 0/2 |  |
| **Hypomagnesemia** |  |  |  |  |  | 2/13 | 0/3 |  |  |  |  |
| **Failure to thrive** |  |  | 0/2 |  |  |  |  |  |  | 2/2 |  |
| **Nephrolithiasis** | 1/3 |  |  |  |  | 5/17 |  | 4/20 | 1/4 |  |  |
| **Hyponatremia** |  |  |  |  |  |  |  |  |  | 0/2 |  |
| **Hypochloremia** |  |  |  |  |  |  |  |  |  | 0/2 |  |
| **Hypouricemia** |  |  |  |  |  |  |  | 0/9 |  |  |  |
| **Metabolic alkalosis** |  |  |  |  |  |  |  |  | 0/4 |  |  |
| **Metabolic acidosis** |  |  |  |  |  |  |  |  | 0/4 |  |  |
| **Bone disorders** |  | 0/6 | 0/2 | 3/5 |  | 5/13 | 0/3 | 9/20 | 2/4 |  | 2/7 |
| **Hypertension** |  |  |  |  |  |  |  |  |  |  |  |
| **Intellectual disability** |  |  |  |  |  |  |  |  |  |  |  |
| **Cataract** |  |  |  |  |  |  |  |  |  |  |  |

LMWP: Low-molecular-weight proteinuria; *already reported in Bökenkamp

| **Clinical sign** | (Sheffer-Babila et al. 2008) | (Cho et al. 2008) | (Bökenkamp et al. 2009, p. 2009) | (Li and Huang 2009) | (Frishberg et al. 2009) | (Becker-Cohen et al. 2012) | (Fervenza 2013) | (Valina et al. 2013) | (Sekine et al. 2014) | (Cramer et al. 2014) | (Mansour-Hendili et al. 2015) |
| --- | --- | --- | --- | --- | --- | --- | --- | --- | --- | --- | --- |
| **n** | 2* | 9 | 212 | 3 | 4 | 7 | 1 | 1 | 61 | 7 | 103 |
| **Population** | American | Korean | Global | Chinese | Israeli | Israeli | American | American | Japanese | American | French |
| **Age (years, range)** | 8-11.5 | 0.2-13 | N/A | 11-12 | 3-11 | 7-35 | 18 | 5 | 0.3-66 | 3-9 | 0.1-55 |
| **Proteinuria** | 2/2 |  |  | 3/3 | 4/4 | 3/7 | 1/1 | 1/1 |  | 7/7 |  |
| **Nephrotic range proteinuria** | 2/2 |  |  |  | 4/4 |  | 1/1 | 1/1 |  | 7/7 |  |
| **LMWP** | * | 9/9 | 212/212 | 3/3 | 4/4 | 7/7 | 1/1 | 1/1 | 61/61 | 4/4 | 99/99 |
| **Hypercalciuria** | * | 9/9 | 180/200 | 3/3 | 1/3 | 5/7 | 1/1 | 1/1 | 25/54 |  | 89/95 |
| **Hyperphosphaturia** | * |  | 35/156 |  | 0/1 | 3/7 |  |  |  |  |  |
| **Aminoaciduria** | * |  | 31/75 |  | 0/3 |  | 0/1 |  |  |  | 23/34 |
| **Glycosuria** | * | 1/9 | 18/108 | 1/3 | 0/4 |  | 0/1 |  |  |  | 27/62 |
| **Nephrocalcinosis** | * |  | 137/182 | 2/3 | 3/4 | 6/7 |  | 0/1 | 20/53 | 2/7 | 59/91 |
| **Renal failure** | * | 0/9 | 60/203 | 0/3 | 0/4 |  |  |  | 4/53 | 4/7 | 52/97 |
| **Hypokalemia** | * |  | 10/67 |  |  | 2/7 |  |  |  | 2/7 | 33/75 |
| **Fanconi incomplete** |  |  |  |  |  |  |  |  |  |  |  |
| **Fanconi complete** |  |  |  |  |  |  |  |  | 0/61 |  |  |
| **Hyperuricosuria** |  | 5/9 |  |  | 0/1 |  | 0/1 |  |  |  |  |
| **Hematuria** | 1/1 | 6/9 |  | 2/3 | 1/2 |  | 1/1 | 0/1 |  |  |  |
| **Hypophosphatemia** | 0/2 | 0/9 |  | 2/3 |  |  | 0/1 | 0/1 |  | 2/7 | 39/65 |
| **Hypocalcemia** |  |  |  | 0/3 |  |  | 0/1 |  |  |  |  |
| **Hypomagnesemia** |  |  |  |  |  |  |  |  |  |  |  |
| **Failure to thrive** | 2/2 |  |  | 1/3 | 0/4 |  |  | 0/1 |  |  |  |
| **Nephrolithiasis** |  |  |  | 0/3 |  |  | 1/1 | 0/1 |  |  | 27/81 |
| **Hyponatremia** |  |  |  |  |  |  |  |  |  |  |  |
| **Hypochloremia** |  |  |  |  |  |  |  |  |  |  |  |
| **Hypouricemia** |  |  |  |  |  |  | 0/1 |  |  |  |  |
| **Metabolic alkalosis** |  |  |  |  |  |  |  |  |  |  |  |
| **Metabolic acidosis** |  | 0/9 | 2/68 |  |  |  |  |  |  |  | 9/60 |
| **Bone disorders** |  | 0/9 |  | 0/3 | 0/1 | 4/7 |  | 0/1 | 0/61 |  | 14/81 |
| **Hypertension** |  |  |  |  |  |  |  |  |  | 0/7 |  |
| **Intellectual disability** |  |  |  |  |  |  |  |  |  |  |  |
| **Cataract** |  |  |  |  |  |  |  |  |  |  |  |

LMWP: Low-molecular-weight proteinuria; *already reported in Bökenkamp

| **Clinical sign** | (Anglani et al. 2015) | (Wang et al. 2016) | (Blanchard et al. 2016) | (Li et al. 2016) | (Zaniew et al. 2017) | (Zhang et al. 2017) | (Wen et al. 2018) | (Ye et al. 2020) | (Bao et al. 2019) | (Deng et al. 2020) | (Sakakibara et al. 2020) |
| --- | --- | --- | --- | --- | --- | --- | --- | --- | --- | --- | --- |
| **n** | 47 | 26 | 108 | 15 | 23 | 6 | 2 | 32 | 3 | 24 | 72 |
| **Population** | Italian | American | French | Chinese | Polish | Chinese | Chinese | Chinese | Chinese | Chinese | Japanese |
| **Age (years, range)** | 1-46 | 1-40 | 5-21 | 3.4-30.6 | 0.3-12.4 | 5-9 | 0.75-1.6 | 0.58-12 |  | 0.3-12.3 | 3.0-9.0 |
| **Proteinuria** |  |  |  | 11/13 | 20/20 |  | 2/2 |  | 3/3 |  |  |
| **Nephrotic range proteinuria** |  |  |  | 5/13 | 3/20 |  | 1/1 |  | 3/3 | 13/24 |  |
| **LMWP** | 46/47 | 20/20 | 93/93 |  | 15/15 | 6/6 | 2/2 | 32/32 | 3/3 | 23/23 | 72/72 |
| **Hypercalciuria** | 43/47 | 17/21 | 81/88 |  | 19/22 | 6/6 | 2/2 | 21/32 | 3/3 | 20/24 | 24/62 |
| **Hyperphosphaturia** | 17/47 |  |  |  | 7/17 |  |  |  |  |  |  |
| **Aminoaciduria** |  |  | 16/32 | 6/15 | 1/11 |  |  |  |  | 6/6 |  |
| **Glycosuria** |  |  | 26/58 | 4/15 | 2/23 |  |  |  |  | 3/24 | 2/68 |
| **Nephrocalcinosis** | 39/47 |  | 44/104 | 1/15 | 13/23 | 3/6 |  | 14/32 |  | 7/22 | 14/63 |
| **Renal failure** | 5/47 | 17/25 |  | 8/15 |  | 0/6 | 0/1 |  |  | 2/20 | 6/71 |
| **Hypokalemia** |  |  | 31/70 | 3/15 | 1/23 |  |  |  |  |  |  |
| **Fanconi incomplete** |  |  | 51/70 |  | 11/23 |  |  |  |  |  |  |
| **Fanconi complete** |  |  | 8/70 |  | 0/23 |  |  |  |  |  |  |
| **Hyperuricosuria** |  |  |  |  |  |  |  |  |  |  |  |
| **Hematuria** |  |  |  | 6/15 |  | 1/6 | 1/2 |  |  | 7/24 |  |
| **Hypophosphatemia** |  |  |  | 5/15 | 9/23 |  |  |  |  | 4/24 |  |
| **Hypocalcemia** |  |  |  | 3/15 |  |  |  |  |  |  |  |
| **Hypomagnesemia** |  |  |  | 5/14 |  |  |  |  |  |  |  |
| **Failure to thrive** |  |  | 12/40 | 5/15 | 5/22 | 1/6 |  |  |  | 4/23 |  |
| **Nephrolithiasis** | 14/47 | 3/24 | 24/74 | 0/15 | 3/23 | 0/6 |  | 3/32 |  | 0/22 |  |
| **Hyponatremia** |  |  |  | 3/15 |  |  |  |  |  |  |  |
| **Hypochloremia** |  |  |  | 3/15 |  |  |  |  |  |  |  |
| **Hypouricemia** |  |  | 19/30 |  | 5/21 |  |  |  |  |  |  |
| **Metabolic alkalosis** |  |  |  | 3/15 |  |  |  |  |  |  |  |
| **Metabolic acidosis** |  |  | 9/54 | 1/15 | 4/22 |  |  |  |  | 0/24 | 0/65 |
| **Bone disorders** | 17/47 | 3/19 | 14/75 |  | 2/23 | 0/6 |  | 3/32 |  | 4/20 |  |
| **Hypertension** |  |  |  |  |  |  |  |  |  |  |  |
| **Intellectual disability** |  |  |  |  |  |  |  |  |  | 2/7 | 5/69 |
| **Cataract** |  |  |  |  |  |  |  |  |  | 1/9 |  |

LMWP: Low-molecular-weight proteinuria

**Supplementary Table 3**: Clinical and biochemical data collected from DD2 patients

| **Clinical sign** | **TOT** | (Hoopes et al. 2005) | (Utsch et al. 2006) | (Sekine et al. 2014) | (Cho et al. 2008) | (Shrimpton et al. 2009) | (Bökenkamp et al. 2009) | (Kaneko et al. 2010) | (Hichri et al. 2011) | (Tasic et al. 2011) | (Becker-Cohen et al. 2012) |
| --- | --- | --- | --- | --- | --- | --- | --- | --- | --- | --- | --- |
| **n** | 143 | 5 | 6 | 4 | 2 | 6 | 28 | 1 | 6 | 3 | 1 |
| **Population** |  | Global | European | Japanese | Korean | Global | Global | Japanese | French | Macedonian | Israeli |
| **Age (years, range)** | 0.1-30.5 | 8-27 | 4.75-12.5 | 3-11 | 0.1-6.9 | 0.5-12 | N/A | 4 | 7-27 | 1.5-10 | 69 |
| **Proteinuria** | 39/39 | - | 6/6 | 4/4 | - | - | - | 1/1 | 1/1 | 3/3 | 1/1 |
| **Nephrotic range proteinuria** | 20/42 | - | 6/6 | 3/4 | - | - | - |  | - | 3/3 | - |
| **LMWP** | 134/134 | 5/5 | 6/6 | 4/4 | 2/2 | 6/6 | 28/28 | 1/1 | 3/3 | 3/3 | 1/1 |
| **Hypercalciuria** | 104/122 | 5/5 | 6/6 |  | 2/2 | 6/6 | 24/28 |  | 4/4 | 2/2 | 1/1 |
| **Hematuria** | 16/32 |  | 3/6 | 1/4 | 2/2 | 2/6 |  | 0/1 |  |  |  |
| **Failure to thrive** | 27/50 | 1/5 | 4/6 |  |  |  |  |  | 0/2 |  |  |
| **Fanconi incomplete** | 10/14 | 5/5 |  |  |  |  |  |  |  |  |  |
| **Renal failure** | 39/89 | 2/5 | 0/6 |  | 0/2 |  | 8/25 |  |  |  |  |
| **Aminoaciduria** | 30/72 | 2/5 | 1/3 | 0/2 |  | 3/6 | 11/21 |  | 1/1 | 1/3 |  |
| **Nephrocalcinosis** | 32/127 | 1/5 | 0/6 | 1/4 |  | 4/6 | 11/28 |  | 1/1 | 0/3 | 0/1 |
| **Hypochloremia** | 1/8 |  |  | 0/4 |  |  |  |  |  |  |  |
| **Hyperphosphaturia** | 9/46 |  |  |  |  |  | 6/25 |  | 2/2 |  | 0/1 |
| **Nephrolithiasis** | 9/66 | 0/5 | 0/6 |  |  | 0/6 |  |  |  | 0/3 |  |
| **Metabolic acidosis** | 6/87 |  | 0/6 | 0/1 | 0/2 |  | 1/27 |  |  |  |  |
| **Bone disorders** | 8/67 | 0/5 | 0/6 | 0/4 | 0/2 | 3/6 |  |  |  |  | 1/1 |
| **Glycosuria** | 11/113 | 0/5 | 1/6 | 0/4 | 0/2 | 1/6 | 3/28 | 0/1 |  | 1/3 |  |
| **Metabolic alkalosis** | 1/10 |  | 0/6 |  |  |  |  |  |  |  |  |
| **Hypokalemia** | 6/56 |  |  | 1/4 |  | 1/6 | 1/18 | 0/1 |  |  | 0/1 |
| **Hypomagnesemia** | 1/19 |  |  |  |  | 0/5 |  |  |  |  |  |
| **Fanconi complete** | 1/38 |  |  |  |  |  |  |  |  |  |  |
| **Hypophosphatemia** | 5/49 | 3/5 | 0/6 |  | 0/2 | 1/6 |  | 0/1 |  |  |  |
| **Hyponatremia** | 0/9 |  |  | 0/4 |  |  |  | 0/1 |  |  |  |
| **Hypocalcemia** | 0/11 |  | 0/6 |  |  |  |  | 0/1 |  |  |  |
| **Hypertension** | 0/19 |  |  |  |  |  |  | 0/1 |  |  |  |
| **Cataract** | 8/87 | 2/5 | 0/6 | 0/4 |  | 1/5 | 2/28 |  | 0/6 | 0/3 |  |
| **Intellectual disability** | 13/53 | 3/5 | 1/6 | 1/4 |  | 3/6 |  |  | 1/4 | 1/3 |  |
| **Behavioral alteration** | 0/20 |  | 0/6 | 0/4 |  |  |  |  |  |  |  |
| **Neurological symptoms** | 4/16 |  |  |  |  |  | 4/6 |  | 0/6 |  |  |
| **Hypotonia** | 1/18 | 0/5 |  |  |  | 1/5 |  |  | 0/3 |  |  |
| **Hyperuricosuria** | 2/2 |  |  |  | 2/2 |  |  |  |  |  |  |
| **Hypouricemia** | 3/5 |  |  | 2/4 |  |  |  |  |  |  |  |

LMWP: Low-molecular-weight proteinuria

| **Clinical sign** | (Sekine et al. 2014) *****1 female | (De Mutiis et al. 2015) | (Blanchard et al. 2016) | (Wang et al. 2016) | (Li et al. 2016) | (Zhang et al. 2017) | (Zaniew et al. 2018) | (Ye et al. 2020) | (Bao et al. 2019) | (Deng et al. 2020) | (Sakakibara et al. 2020) |
| --- | --- | --- | --- | --- | --- | --- | --- | --- | --- | --- | --- |
| **n** | 11 | 1 | 9 | 2 | 4 | 1 | 18 | 13 | 3 | 7 | 13 |
| **Population** | Japanese | Italian | French | American | Chinese | Chinese | Global | Chinese | Chinese | Chinese | Japanese |
| **Age (years, range)** | 0.3-24.8 | 2 | 3-8 | 7 | 5.6-30.5 | 6 | 7-17 | 0.17-10 |  | 0.2-7.2 | 1.5-5.5 |
| **Proteinuria** | - | 1/1 | - | - | 2/2 |  | 17/17 | - | 3/3 | - |  |
| **Nephrotic range proteinuria** | - | 1/1 | - | - | 1/1 |  | 0/17 | - | 3/3 | 3/7 |  |
| **LMWP** | 11/11 | 1/1 | 7/7 | 2/2 |  | 1/1 | 17/17 | 13/13 | 3/3 | 7/7 | 13/13 |
| **Hypercalciuria** | 7/10 | 0/1 | 3/3 | 2/2 |  | 1/1 | 12/18 | 12/13 | 3/3 | 7/7 | 7/10 |
| **Hematuria** |  | 1/1 |  |  | 4/4 | 1/1 |  |  |  | 3/7 |  |
| **Failure to thrive** |  | 0/1 | 4/6 |  | 4/4 | 0/1 | 9/18 |  |  | 5/7 |  |
| **Fanconi incomplete** |  |  | 5/9 |  |  |  |  |  |  |  |  |
| **Renal failure** | 0/10 | 0/1 |  | 2/2 |  | 0/1 | 18/18 |  |  | 2/7 | 7/12 |
| **Aminoaciduria** |  | 0/1 | 4/5 |  | 0/4 |  | 4/18 |  |  | 3/3 |  |
| **Nephrocalcinosis** | 1/10 | 0/1 | 1/9 |  | 0/4 | 0/1 | 9/18 | 2/13 |  | 1/5 | 0/12 |
| **Hypochloremia** |  |  |  |  | 1/4 |  |  |  |  |  |  |
| **Hyperphosphaturia** |  |  |  |  |  |  | 1/18 |  |  |  |  |
| **Nephrolithiasis** |  | 0/1 | 1/6 | 0/2 | 0/4 | 0/1 | 6/18 | 2/13 |  | 0/5 |  |
| **Metabolic acidosis** |  | 0/1 | 2/8 |  | 0/4 |  | 3/18 |  |  | 0/7 | 0/13 |
| **Bone disorders** | 1/11 |  | 1/7 |  | 0/4 | 0/1 |  | 2/13 |  | 0/7 |  |
| **Glycosuria** |  | 0/1 | 0/6 |  | 0/4 |  | 4/18 |  |  | 0/7 | 0/13 |
| **Metabolic alkalosis** |  |  |  |  | 1/4 |  |  |  |  |  |  |
| **Hypokalemia** |  |  | 1/4 |  | 1/4 |  | 1/18 |  |  |  |  |
| **Hypomagnesemia** |  |  |  |  | 0/4 |  | 1/10 |  |  |  |  |
| **Fanconi complete** | 0/11 |  | 1/9 |  |  |  | 0/18 |  |  |  |  |
| **Hypophosphatemia** |  |  |  |  | 0/4 |  | 0/18 |  |  | 1/7 |  |
| **Hyponatremia** |  |  |  |  | 0/4 |  |  |  |  |  |  |
| **Hypocalcemia** |  |  |  |  | 0/4 |  |  |  |  |  |  |
| **Hypertension** |  |  |  |  |  |  | 0/18 |  |  |  |  |
| **Cataract** | 1/6 | 0/1 |  |  | 0/4 |  | 0/6 |  | 0/3 | 2/3 | 0/13 |
| **Intellectual disability** | 0/7 | 0/1 |  |  | 0/4 |  |  |  | 0/3 | 0/1 | 3/13 |
| **Behavioral alteration** | 0/7 |  |  |  |  |  |  |  | 0/3 |  |  |
| **Neurological symptoms** |  |  |  |  |  |  | 0/4 |  |  |  |  |
| **Hypotonia** |  | 0/1 |  |  | 0/4 |  |  |  |  |  |  |
| **Hyperuricosuria** |  |  |  |  |  |  |  |  |  |  |  |
| **Hypouricemia** |  |  | 1/1 |  |  |  |  |  |  |  |  |

LMWP: Low-molecular-weight proteinuria

**Supplementary Table 4**: Clinical and biochemical data collected from Dent disease patients without *CLCN5* and *OCRL* mutations (DD3)

| **Clinical sign** | **TOT** | (Hoopes et al. 2004, 2005) | (Utsch et al. 2006) | (Sekine et al. 2007) | (Ramos-Trujillo et al. 2007b) | (Cho et al. 2008) | (Saida et al. 2014) *****1 female | (Anglani et al. 2015) | (Wang et al. 2016) | (Zhang et al. 2017) |
| --- | --- | --- | --- | --- | --- | --- | --- | --- | --- | --- |
| **n** | 64 | 8 | 14 | 5 | 2 | 1 | 14 | 17 | 2 | 1 |
| **Population** |  | Global | European | Japanese | Spanish | Korean | Japanese | Italian | Global | Chinese |
| **Age (years, range)** | 2-58 | 4-17 | 2-51 | - | - | 3.5 | - | 2-58 | 38-48 | 8 |
| **LMWP** | 64/64 | 8/8 | 14/14 | 5/5 | 2/2 | 1/1 | 14/14 | 17/17 | 2/2 | 1/1 |
| **Hypercalciuria** | 44/59 | 8/8 | 11/14 | 3/5 | 2/2 | 1/1 | 5/9 | 12/17 | 1/2 | 1/1 |
| **Hematuria** | 1/7 |  |  | 0/5 |  | 1/1 |  |  |  | 1/1 |
| **Renal failure** | 13/58 | 2/7 | 2/14 | 0/5 |  | 1/1 | 2/11 | 4/17 | 2/2 | 0/1 |
| **Aminoaciduria** | 3/5 | 3/5 |  |  |  |  |  |  |  |  |
| **Hyperphosphaturia** | 9/17 |  |  |  |  |  |  | 9/17 |  |  |
| **Bone disorders** | 10/54 | 3/6 | 1/14 |  |  |  | 1/14 | 5/17 | 0/2 | 0/1 |
| **Glycosuria** | 5/7 | 4/6 |  |  |  | 1/1 |  |  |  |  |
| **Fanconi complete** | 0/14 |  |  |  |  |  | 0/14 |  |  |  |
| **Hypophosphatemia** | 6/13 | 4/7 |  | 1/5 |  | 1/1 |  |  |  |  |
| **Nephrocalcinosis** | 19/34 | 7/7 |  |  |  |  | 5/9 | 7/17 |  | 0/1 |
| **Nephrolithiasis** | 8/27 | 1/7 |  |  |  |  |  | 5/17 | 2/2 | 0/1 |
| **Cataract** | 0/5 |  |  | 0/5 |  |  |  |  |  |  |
| **Intellectual disability** | 0/5 |  |  | 0/5 |  |  |  |  |  |  |
| **Behavioral alteration** | 0/5 |  |  | 0/5 |  |  |  |  |  |  |
| **Neurological symptoms** | 0/1 |  |  |  |  | 0/1 |  |  |  |  |
| **Hypotonia** | 0/5 |  |  | 0/5 |  |  |  |  |  |  |
| **Hyperuricosuria** | 1/1 |  |  |  |  | 1/1 |  |  |  |  |

LMWP: Low-molecular-weight proteinuria

**Supplementary table 5:** Kidney biopsies of Dent disease patients. Nucleotide numbering is according to the *CLCN5* cDNA sequence (GenBank entry NM_000084.4). The A of the ATG of the Methionine initiation codon is defined as nucleotide 1.

| **Dent disease type** | **Mutation** | | **Histology** | | **TEM** | **IF** | **Ref** |
| --- | --- | --- | --- | --- | --- | --- | --- |
|  | **Nucleotide** | **Protein** | **Glomerular** | **Interstitial** |  |  |  |
| 1 | c.394-2A>C |  | Glomerulosclerosis | Tubular atrophy, No calcification | N/A | N/A | (Igarashi et al. 1998) |
| 1 | c.726+1G>A |  | FSGS, Focal glomerular obsolescence, Periglomerular thickening | Interstitial fibrosis, Tubular atrophy, Interstitial mononuclear cell infiltration | Foot processes effacement | Negative | (Kubo et al. 2016) |
| 1 |  | p.Arg28* | Glomerulosclerosis | Interstitial fibrosis, Calcification | N/A | N/A | (Hoopes et al. 1998) |
| 1 | c.82C>T | p.Arg28* | FSGS |  | Normal | Normal | (Zaniew et al. 2017) |
| 1 | c.92delA | p.Val31fs*15 | Global sclerosis, No mesangial expansion or capillary wall deposits | Cortical fibrosis, Interstitial calcification, Calcium phosphate deposits | Negligible foot process effacement | Nonspecific IgM mesangial staining | (Fervenza 2013) |
| 1 |  | p.Arg34* | Glomerulosclerosis | Interstitial fibrosis, Tubular atrophy | N/A | N/A | (Hoopes et al. 1998) |
| 1 |  | p.Arg34* | Focal periglomerular fibrosis, Focal glomerulosclerosis | Interstitial fibrosis, Tubular atrophy, Interstitial lymphocytic infiltrate | Normal GBM, No immune deposits | N/A | (Langlois et al. 1998) |
| 1 | c.310C>T | p.Arg104* | FSGS, FGGS, Mesangial proliferation, Extracellular matrix hyperplasia | Intratubular protein casts, Vascular degeneration | Mesangial proliferation with less electron dense deposits | Negative | (Wen et al. 2018) |
| 1 |  | p.Glu118* | Normal | Normal | N/A | N/A | (Morimoto et al. 1998) |
| 1 | c.366G>A | p.Trp122* | FGGS, FSGS, Podocyte hypertrophy, Collapse of the glomerular tuft | Mild interstitial fibrosis, foci of tubular atrophy | Collapsed glomerulus (only 1 analyzed) | Negative | (Frishberg et al. 2009) |
| 1 | Exon 5 deletion |  | Glomerulosclerosis, Mesangial proliferation | Tubular atrophy, Intratubular proteinaceous casts, No calcification | N/A | Negative | (Brakemeier et al. 2004) |
| 1 |  | p.Asp130Gly | FSGS, FGGS | Tubular atrophy and dilatation | N/A | N/A | (Saida et al. 2014) |
| **Dent disease type** | **Mutation** | | **Histology** | | **TEM** | **IF** | **Ref** |
|  | **Nucleotide** | **Protein** | **Glomerular** | **Interstitial** |  |  |  |
| 1 | c.458_459insA | p.Thr153fs*15 | Mesangial proliferative glomerulonephritis |  | N/A | N/A | (Bao et al. 2019) |
| 1 | c.483delA | p.Gly163Aspfs*45 | Mesangial proliferative glomerulonephritis |  | N/A | N/A | (Deng et al. 2020) |
| 1 | c.509_510insA | p.His170fs*7 | Immature glomeruli | Occasional calcium deposits | N/A | N/A | (Matsuyama et al. 2004) |
| 1 | c.518delG | p.Trp173* | Mesangial hypercellularity | Focal interstitial inflammation, Tubular damage | Foot process effacement | N/A | (Zhang et al. 2017) |
| 1 | c.523dupA | p.Thr175fs*8 | FGGS, Perihiliar hyalinosis, Minimal segmental increase in mesangial cells | Slight tubular atrophy and interstitial fibrosis | Foot process effacement without electron-dense deposits, normal GBM | Negative | (Copelovitch et al. 2007) |
| 1 | c.965T>C | p.Leu225Pro | Normal | Normal | N/A | N/A | (Ramos-Trujillo et al. 2007a) |
| 1 | c.744_746dupACG | p.249:insAla | FGGS, FSGS, Periglomerular fibrosis | Mild interstitial chronic inflammation, Intratubular proteinaceous casts | Foot process effacement without electron-dense deposits, Thickness of the GBM | Sample unsuitable for analysis | (Copelovitch et al. 2007) |
| 1 | c.782G>T | p.Gly261Val | Normal | Normal | Foot process effacement, MCD | N/A | (Zaniew et al. 2017) |
| 1 § |  | p.Ser270Arg | Glomerulosclerosis | Calcification, Tubular atrophy | N/A | N/A | (Igarashi et al. 1998) |
| 1 § |  | p.Ser270Arg | Glomerulosclerosis | Calcification, Tubular atrophy | N/A | N/A | (Igarashi et al. 1998) |
| 1 | c.815A>G | p.Tyr272Cys | Normal | Normal | Normal | Negative | (Hellemans et al. 2010) |
| 1 |  | p.Leu278Phe | Glomerulosclerosis | Tubular atrophy, No calcification | N/A | N/A | (Igarashi et al. 1998) |
| 1 | c.882dupC | p.Ile295fs*24 | FGGS, Immature glomeruli | Tubular atrophy | Foot process effacement | Negative | (Sethi et al. 2009) |
| **Dent disease type** | **Mutation** | | **Histology** | | **TEM** | **IF** | **Ref** |
|  | **Nucleotide** | **Protein** | **Glomerular** | **Interstitial** |  |  |  |
| 1 | c.940delT | p.Ser314Argfs*10 | Mesangial proliferative glomerulonephritis |  | N/A | N/A | (Deng et al. 2020) |
| 1 |  | p.Gly329Asp | Focal glomerulosclerosis, Mesangial proliferation, Adherence to Bowman capsule | Interstitial fibrosis, Tubular atrophy | N/A | IgM | (Vaisbich et al. 2012) |
| 1 | c.989G>A | p.Gly30Asp | Mild glomerular lesions | Tubular injury | N/A | N/A | (Deng et al. 2020) |
| 1 | c.992_993insAGTATTAT | p.Pro34fs*1 | Mild glomerular lesions |  | N/A | N/A | (Deng et al. 2020) |
| 1 | c.997G>A | p.Gly333Arg | FSGS |  | Foot process effacement | Negative | (Zaniew et al. 2017) |
| 1 § | c.1200delC | p.Leu400fs*33 | Mesangial proliferation |  | Foot process effacement | N/A | (Zaniew et al. 2017) |
| 1 § | c.1200delC | p.Leu400fs*33 | FSGS |  | N/A | N/A | (Zaniew et al. 2017) |
| 1 | c.1396G > C | p.Gly466Arg | FGGS, FSGS, Periglomerular fibrosis | Interstitial fibrosis, Tubular atrophy | Foot process effacement, Normal GBM | Negative | (Valina et al. 2013) |
| 1 | c.1413_1414delTG | p.Cys471* | Mesangial proliferative glomerulonephritis |  | N/A | N/A | (Deng et al. 2020) |
| 1 | c.1444delG | p.Gly483Valfs*21 | Mild glomerular lesions |  | N/A | N/A | (Deng et al. 2020) |
| 1 | c.1647C>A | p.Tyr549* | FGGS | Focal interstitial fibrosis, Focal interstitial inflammation, Tubular damage, Nephrocalcinosis | Foot process effacement | N/A | (Zhang et al. 2017) |
| 1 |  | p.Ile524Lys | Glomerulosclerosis | Tubulointerstitial fibrosis, Tubular atrophy, Calcium deposits |  | Negative | (Yanagida et al. 2004) |
| 1 § | c.1618G>C | p.Ala540Pro | FSGS | Normal | N/A | N/A | (Wong et al. 2017) |
| 1 § | c.1618G>C | p.Ala540Pro | Normal | Tubular calcification, Cast material in the tubular lumen | N/A | N/A | (Wong et al. 2017) |
| **Dent disease type** | **Mutation** | | **Histology** | | **TEM** | **IF** | **Ref** |
|  | **Nucleotide** | **Protein** | **Glomerular** | **Interstitial** |  |  |  |
| 1 § | c.1618G>C | p.Ala540Pro | Normal | Tubular calcification, Cast material in the tubular lumen | N/A | N/A | (Wong et al. 2017) |
| 1 § | c.1618G>C | p.Ala540Pro | Normal | Tubular calcifrication, Cast material in the tubular lumen | N/A | N/A | (Wong et al. 2017) |
| 1 |  | p.Tyr567* | Focal glomerulosclerosis | Tubulointerstitial fibrosis, Mononuclear cell infiltration | N/A | Negative | (Okamoto et al. 2012) |
| 1 | c.1711C>T | p.Leu571Phe | Mesangial proliferative glomerulonephritis |  | N/A | N/A | (Bao et al. 2019) |
| 1 | c.1720A>G | p.Met574Val | Mild glomerular lesions |  | N/A | N/A | (Deng et al. 2020) |
| 1 | c.1726G>A | p.Gly576Arg | FGGS | Normal | N/A | N/A | (Matsuyama et al. 2004) |
| 1 | c.1745-2A>G | - | Mild glomerular lesions | Tubular interstitial nephritis | N/A | N/A | (Deng et al. 2020) |
| 1 § | c.1771C>T | p.Leu591Phe | FSGS |  | N/A | N/A | (Solanki et al. 2018) |
| 1 § | c.1771C>T | p.Leu591Phe | FSGS |  | N/A | N/A | (Solanki et al. 2018) |
| 1 | c.1799G>T | p.Gly600Val | FGGS, Mesangial hypercellularity | Focal interstitial inflammation, Tubular damage, Nephrocalcinosis | Foot process effacement | N/A | (Bao et al. 2019) |
| 1 | c.1843A>C | p.Ser615Arg | FGGS | Focal interstitial inflammation, Tubular damage, Nephrocalcinosis | Foot process effacement | N/A | (Zhang et al. 2017) |
| 1 | c.1889-1890delC | p.His631Thrfs*25 | Normal | Normal | N/A | N/A | (Deng et al. 2020) |
| 1 |  | p.Arg637* | Mesangial proliferation | Calcium crystal deposition | N/A | Negative | (Takemura et al. 2001) |
| 1 |  | p.Arg637* | Normal | Focal medullary calcification | N/A | N/A | (Cheong et al. 2005) |
| 1 |  | p.Arg648* | FGGS, Mesangial proliferation without mesangial matrix expansion | Normal | Sample unsuitable for analysis | Negative | (Frishberg et al. 2009) |
| 1 § |  | P.Arg648* | Normal | Calcification | N/A | N/A | (Igarashi et al. 1998) |
| **Dent disease type** | **Mutation** | | **Histology** | | **TEM** | **IF** | **Ref** |
|  | **Nucleotide** | **Protein** | **Glomerular** | **Interstitial** |  |  |  |
| 1 § |  | P.Arg648* | Glomerulosclerosis | Calcification | N/A | N/A | (Igarashi et al. 1998) |
| 1 | c.1975delC | p.Arg659Glyfs*7 | FSGS | Chronic tubulointerstitial damage | N/A | N/A | (Deng et al. 2020) |
| 1 |  | p.Arg704* | Glomerulosclerosis, Periglomerular fibrosis | Interstitial fibrosis, Tubular atrophy, Tubulointerstitial calcium deposits, Nephrocalcinosis | Normal GBM | Negative | (Langlois et al. 1998) |
| 1 | c.2119C>T | p.Arg707* |  | Mild tubulointerstitial lesions | N/A | N/A | (Li and Huang 2009) |
| 1 | c.2119C>T | p.Arg707* | FGGS, Mesangial hypercellularity | Focal interstitial inflammation, Tubular damage | Normal | N/A | (Zhang et al. 2017) |
| 1 § | c.2179delG | p.Asp727fs*2 | FGGS |  | N/A | N/A | (Sheffer-Babila et al. 2008) |
| 1 § | c.2179delG | p.Asp727fs*2 | FGGS |  | N/A | N/A | (Sheffer-Babila et al. 2008) |
| 1 | c.2179delG | p.Asp727fs | FGGS, FSGS | Normal | Global sclerosis, Foot process effacement | Negative | (Frishberg et al. 2009) |
| 1 | *CLCN5* missense mutations | | Mesangial proliferative glomerulonephritis |  | N/A | N/A | (Bao et al. 2019) |
| 1 | 6 male patients | | Segmental sclerosis 3/6, Segmental increase in mesangial matrix 3/6 | Interstitial calcium phosphate deposits 2/6, Intratubular proteinaceous casts 2/6, Interlobular arteries’ medial thickening 2/6, Intimal arterioles’ proliferation 2/6, tubular hypoplasia 1/6 | Foot process effacement in all the 5 biopsies analyzed | N/A | (Cramer et al. 2014) |
| 1 | N/A | N/A | Global sclerosis | Interstitial fibrosis | Normal | Nonspecific IgM and C3 deposition | (Anglani et al. 2006) |
| 1 | N/A | N/A | FSGS |  | N/A | N/A | (Becker-Cohen et al. 2012) |
| **Dent disease type** | **Mutation** | | **Histology** | | **TEM** | **IF** | **Ref** |
|  | **Nucleotide** | **Protein** | **Glomerular** | **Interstitial** |  |  |  |
| 1 | N/A | N/A | Normal | Normal | N/A | N/A | (Deng et al. 2020) |
| 1 | N/A | N/A | Mesangial proliferative glomerulonephritis | Tubulointerstitial nephritis | N/A | N/A | (Deng et al. 2020) |
| 1 | N/A | N/A | Normal | Normal | N/A | N/A | (Deng et al. 2020) |
| 1 | N/A | N/A | Mild glomerular lesions | Chronic tubulointerstitial nephropathy with ischemic renal damage | N/A | N/A | (Deng et al. 2020) |
| 1 | N/A | N/A | Mild glomerular lesions |  | N/A | N/A | (Deng et al. 2020) |
| 2 |  | p.Gln87fs*18 | FGGS | Normal | Foot process effacement | N/A | (Zhang et al. 2017) |
| 2 | c.269G>A | p.Trp90* | Mild glomerular lesions |  |  |  | (Deng et al. 2020) |
| 2 | c.310_313delTGTT | p.Cys104* | Mesangial proliferative glomerulonephritis |  | N/A | N/A | (Bao et al. 2019) |
| 2 | c.547delT | p.Ser183fs*2 | Mesangial proliferative glomerulonephritis |  | N/A | N/A | (Bao et al. 2019) |
| 2 |  | p.Arg301Cys | Minor glomerular abnormalities |  | N/A | N/A | (Sekine et al. 2007) |
| 2 | c.952C>T | p.Arg318Cys | Minor glomerular abnormalities |  | N/A | N/A | (De Mutiis et al. 2015) |
| 2 | c.1110C>G | p.Cys370Trp | Mesangial proliferative glomerulonephritis | Tubular injury | N/A | N/A | (Deng et al. 2020) |
| 2 § | c1467-2A>G |  | FSGS, Adherence of the glomerular tuft to the Bowman’s capsule, Mesangial hypercellularity, Increased mesangial matrix deposition | Normal tubular morphology, Proteinaceous material in the tubules, Interstitial expansion without fibrosis | Foot process effacement, Irregular in-folding of the GBM | Negative | (Preston et al. 2020) |
| 2 § | c1467-2A>G |  | Normal | Acute tubular necrosis | N/A | N/A | (Preston et al. 2020) |
| 2 | c.1525C>T | p.Pro509Ser | FSGS, Mesangial proliferation | Focal tubular atrophy, Focal fibrosis, Lymphocytes infiltration | N/A | N/A | (Kaneko et al. 2010) |
| **Dent disease type** | **Mutation** | | **Histology** | | **TEM** | **IF** | **Ref** |
|  | **Nucleotide** | **Protein** | **Glomerular** | **Interstitial** |  |  |  |
| 2 | c.1567G>A | p.Asp523Asn | Mesangial proliferative glomerulonephritis |  | N/A | N/A | (Bao et al. 2019) |
| 2 | c.2435T>C | p.Leu81Pro | Mild glomerular lesions |  | N/A | N/A | (Deng et al. 2020) |
| 2 | N/A | N/A | Normal | Normal | N/A | Negative | (Tasic et al. 2011) |
| 2 | N/A | N/A | Mesangial proliferative glomerulonephritis |  | N/A | N/A | (Deng et al. 2020) |
| 2 | N/A | N/A | Normal | Normal | N/A | N/A | (Deng et al. 2020) |
| 2 | N/A | N/A | Normal | Normal | N/A | N/A | (Deng et al. 2020) |
| 2 | N/A | N/A | FSGS |  | N/A | N/A | (Deng et al. 2020) |
| 1/2/3 | 30 different subjects | | FGGS 25/30, Mesangial hypercellularity 4/30, FSGS 2/30, Focal segmental glomerular capillary collapse 2/30, Cellular crescent 1/30 | Tubular damage 21/30, Focal interstitial fibrosis 18/30, Focal interstitial inflammation 16/30, Nephrocalcinosis 6/30 | Foot process effacement 13/23 | Negative 30/30 | (Wang et al. 2016) |
| ** |  |  | Glomerulosclerosis | Tubular and interstitial calcium deposits, Interstitial fibrosis, Focal tubular atrophy | Normal GBM | Negative | (Langlois et al. 1998) |
| ? § |  |  | Glomerulosclerosis | Interstitial fibrosis, Tubular atrophy, Nonspecific interstitial lymphocytic infiltration, Calcium deposition | N/A | N/A | (Frymoyer et al. 1991) |
| ? § |  |  | FSGS, FGGS, Immature glomeruli | Normal | Foot process effacement in sclerotic glomeruli, Capillary basement membranes thickness | Negative | (Frymoyer et al. 1991) |
| ? | 3 unrelated male patients | | Periglomerular fibrosis, Partial or complete hyalinization of some glomeruli | Chronic interstitial nephritis, Interstitial fibrosis, Tubular atrophy, Dilatation and cast formation, Calcification | N/A | Negative | (Wrong et al. 1994) |
| **Dent disease type** | **Mutation** | | **Histology** | | **TEM** | **IF** | **Ref** |
|  | **Nucleotide** | **Protein** | **Glomerular** | **Interstitial** |  |  |  |
| ? |  |  | Minor glomerular abnormalities | Focal area of peritubular calcification | N/A | Negative | (Igarashi et al. 1995) |
| ? |  |  | Global glomerulosclerosis | Tubular atrophy, focal area of peritubular calcification | N/A | Negative | (Igarashi et al. 1995) |

TEM: transmission electron microscopy, IF: immunofluorescence, FSGS: focal segmental glomerulosclerosis, FGGS: focal global glomerulosclerosis, MCD: minimal change disease, GBM: glomerular basement membrane, N/A: not available data, §: related subjects, ?: genetic analysis not performed, **: DD1 excluded but only *CLCN5* gene analysis was performed.

**References**

Akuta N, Lloyd SE, Igarashi T, et al (1997) Mutations of CLCN5 in Japanese children with idiopathic low molecular weight proteinuria, hypercalciuria and nephrocalcinosis. Kidney Int 52:911–916. https://doi.org/10.1038/ki.1997.412

Anglani F, Bernich P, Tosetto E, et al (2006) Family history may be misleading in the diagnosis of Dent’s disease. Urol Res 34:61–63. https://doi.org/10.1007/s00240-005-0005-5

Anglani F, D’Angelo A, Bertizzolo LM, et al (2015) Nephrolithiasis, kidney failure and bone disorders in Dent disease patients with and without CLCN5 mutations. Springerplus 4:492. https://doi.org/10.1186/s40064-015-1294-y

Bao Y, Suo L, Qian P, et al (2019) Clinical and genetic analysis of Dent disease with nephrotic range albuminuria in Shaanxi, China. Sci China Life Sci 62:1590–1593. https://doi.org/10.1007/s11427-018-9829-0

Becker-Cohen R, Rinat C, Ben-Shalom E, et al (2012) Vitamin A deficiency associated with urinary retinol binding protein wasting in Dent’s disease. Pediatr Nephrol 27:1097–1102. https://doi.org/10.1007/s00467-012-2121-0

Bignon Y, Alekov A, Frachon N, et al (2018) A novel CLCN5 pathogenic mutation supports Dent disease with normal endosomal acidification. Hum Mutat 39:1139–1149. https://doi.org/10.1002/humu.23556

Blanchard A, Curis E, Guyon-Roger T, et al (2016) Observations of a large Dent disease cohort. Kidney International 90:430–439. https://doi.org/10.1016/j.kint.2016.04.022

Bökenkamp A, Böckenhauer D, Cheong HI, et al (2009) Dent-2 Disease: A Mild Variant of Lowe Syndrome. The Journal of Pediatrics 155:94–99. https://doi.org/10.1016/j.jpeds.2009.01.049

Brakemeier S, Si H, Gollasch M, et al (2004) Dent’s disease: identification of a novel mutation in the renal chloride channel CLCN5. Clin Nephrol 62:387–390. https://doi.org/10.5414/cnp62387

Carballo-Trujillo I, Garcia-Nieto V, Moya-Angeler FJ, et al (2003) Novel truncating mutations in the ClC-5 chloride channel gene in patients with Dent’s disease. Nephrol Dial Transplant 18:717–723. https://doi.org/10.1093/ndt/gfg016

Carr G, Simmons N, Sayer J (2003) A role for CBS domain 2 in trafficking of chloride channel CLC-5. Biochem Biophys Res Commun 310:600–605. https://doi.org/10.1016/j.bbrc.2003.09.057

Chang M-H, Brown MR, Liu Y, et al (2020) Cl- and H+ coupling properties and subcellular localizations of wildtype and disease-associated variants of the voltage-gated Cl-/H+ exchanger ClC-5. J Biol Chem 295:1464–1473. https://doi.org/10.1074/jbc.RA119.011366

Cheong HI, Lee JW, Zheng SH, et al (2005) Phenotype and genotype of Dent’s disease in three Korean boys. Pediatr Nephrol 20:455–459. https://doi.org/10.1007/s00467-004-1769-5

Cho HY, Lee BH, Choi HJ, et al (2008) Renal manifestations of Dent disease and Lowe syndrome. Pediatr Nephrol 23:243–249. https://doi.org/10.1007/s00467-007-0686-9

Copelovitch L, Nash MA, Kaplan BS (2007) Hypothesis: Dent disease is an underrecognized cause of focal glomerulosclerosis. Clin J Am Soc Nephrol 2:914–918. https://doi.org/10.2215/CJN.00900207

Cox JP, Yamamoto K, Christie PT, et al (1999) Renal chloride channel, CLCN5, mutations in Dent’s disease. J Bone Miner Res 14:1536–1542. https://doi.org/10.1359/jbmr.1999.14.9.1536

Cramer MT, Charlton JR, Fogo AB, et al (2014) Expanding the phenotype of proteinuria in Dent disease. A case series. Pediatr Nephrol 29:2051–2054. https://doi.org/10.1007/s00467-014-2824-5

D’Antonio C, Molinski S, Ahmadi S, et al (2013) Conformational defects underlie proteasomal degradation of Dent’s disease-causing mutants of ClC-5. Biochem J 452:391–400. https://doi.org/10.1042/BJ20121848

De Mutiis C, Pasini A, La Scola C, et al (2015) Nephrotic-range Albuminuria as the presenting symptom of Dent-2 disease. Ital J Pediatr 41:46. https://doi.org/10.1186/s13052-015-0152-4

Deng H, Zhang Y, Xiao H, et al (2020) Phenotypic spectrum and antialbuminuric response to angiotensin converting enzyme inhibitor and angiotensin receptor blocker therapy in pediatric Dent disease. Mol Genet Genomic Med e1306. https://doi.org/10.1002/mgg3.1306

Fervenza FC (2013) A patient with nephrotic-range proteinuria and focal global glomerulosclerosis. Clin J Am Soc Nephrol 8:1979–1987. https://doi.org/10.2215/CJN.03400313

Friedrich T, Breiderhoff T, Jentsch TJ (1999) Mutational analysis demonstrates that ClC-4 and ClC-5 directly mediate plasma membrane currents. J Biol Chem 274:896–902. https://doi.org/10.1074/jbc.274.2.896

Frishberg Y, Dinour D, Belostotsky R, et al (2009) Dent’s disease manifesting as focal glomerulosclerosis: Is it the tip of the iceberg? Pediatr Nephrol 24:2369–2373. https://doi.org/10.1007/s00467-009-1299-2

Frymoyer PA, Scheinman SJ, Dunham PB, et al (1991) X-linked recessive nephrolithiasis with renal failure. N Engl J Med 325:681–686. https://doi.org/10.1056/NEJM199109053251003

Gorvin CM, Wilmer MJ, Piret SE, et al (2013) Receptor-mediated endocytosis and endosomal acidification is impaired in proximal tubule epithelial cells of Dent disease patients. Proc Natl Acad Sci USA 110:7014–7019. https://doi.org/10.1073/pnas.1302063110

Grand T, L’Hoste S, Mordasini D, et al (2011) Heterogeneity in the processing of CLCN5 mutants related to Dent disease. Hum Mutat 32:476–483. https://doi.org/10.1002/humu.21467

Grand T, Mordasini D, L’Hoste S, et al (2009) Novel CLCN5 mutations in patients with Dent’s disease result in altered ion currents or impaired exchanger processing. Kidney Int 76:999–1005. https://doi.org/10.1038/ki.2009.305

Hellemans R, Verpooten GA, Bosmans J-L (2010) A young man presenting with recurrent nephrolithiasis. NDT Plus 3:584–587. https://doi.org/10.1093/ndtplus/sfq161

Hichri H, Rendu J, Monnier N, et al (2011) From Lowe syndrome to Dent disease: correlations between mutations of the OCRL1 gene and clinical and biochemical phenotypes. Hum Mutat 32:379–388. https://doi.org/10.1002/humu.21391

Hoopes RR, Hueber PA, Reid RJ, et al (1998) CLCN5 chloride-channel mutations in six new North American families with X-linked nephrolithiasis. Kidney Int 54:698–705. https://doi.org/10.1046/j.1523-1755.1998.00061.x

Hoopes RR, Raja KM, Koich A, et al (2004) Evidence for genetic heterogeneity in Dent’s disease. Kidney Int 65:1615–1620. https://doi.org/10.1111/j.1523-1755.2004.00571.x

Hoopes RR, Shrimpton AE, Knohl SJ, et al (2005) Dent Disease with mutations in OCRL1. Am J Hum Genet 76:260–267. https://doi.org/10.1086/427887

Igarashi T, Günther W, Sekine T, et al (1998) Functional characterization of renal chloride channel, CLCN5, mutations associated with Dent’sJapan disease. Kidney Int 54:1850–1856. https://doi.org/10.1046/j.1523-1755.1998.00203.x

Igarashi T, Hayakawa H, Shiraga H, et al (1995) Hypercalciuria and nephrocalcinosis in patients with idiopathic low-molecular-weight proteinuria in Japan: is the disease identical to Dent’s disease in United Kingdom? Nephron 69:242–247. https://doi.org/10.1159/000188464

Igarashi T, Inatomi J, Ohara T, et al (2000) Clinical and genetic studies of CLCN5 mutations in Japanese families with Dent’s disease. Kidney Int 58:520–527. https://doi.org/10.1046/j.1523-1755.2000.00198.x

Kaneko K, Hasui M, Hata A, et al (2010) Focal segmental glomerulosclerosis in a boy with Dent-2 disease. Pediatr Nephrol 25:781–782. https://doi.org/10.1007/s00467-009-1362-z

Kelleher CL, Buckalew VM, Frederickson ED, et al (1998) CLCN5 mutation Ser244Leu is associated with X-linked renal failure without X-linked recessive hypophosphatemic rickets. Kidney Int 53:31–37. https://doi.org/10.1046/j.1523-1755.1998.00752.x

Kubo K, Aizawa T, Watanabe S, et al (2016) Does Dent disease remain an underrecognized cause for young boys with focal glomerulosclerosis? Pediatr Int 58:747–749. https://doi.org/10.1111/ped.12944

Langlois V, Bernard C, Scheinman SJ, et al (1998) Clinical features of X-linked nephrolithiasis in childhood. Pediatr Nephrol 12:625–629. https://doi.org/10.1007/s004670050516

Li F, Yue Z, Xu T, et al (2016) Dent Disease in Chinese Children and Findings from Heterozygous Mothers: Phenotypic Heterogeneity, Fetal Growth, and 10 Novel Mutations. J Pediatr 174:204-210.e1. https://doi.org/10.1016/j.jpeds.2016.04.007

Li P, Huang J-P (2009) Phenotype and genotype of Dent’s disease in three Chinese boys. Nephrology (Carlton) 14:139–142. https://doi.org/10.1111/j.1440-1797.2008.01057.x

Lloyd SE, Gunther W, Pearce SH, et al (1997a) Characterisation of renal chloride channel, CLCN5, mutations in hypercalciuric nephrolithiasis (kidney stones) disorders. Hum Mol Genet 6:1233–1239. https://doi.org/10.1093/hmg/6.8.1233

Lloyd SE, Pearce SH, Günther W, et al (1997b) Idiopathic low molecular weight proteinuria associated with hypercalciuric nephrocalcinosis in Japanese children is due to mutations of the renal chloride channel (CLCN5). J Clin Invest 99:967–974. https://doi.org/10.1172/JCI119262

Lloyd SE, Pearce SHS, Fisher SE, et al (1996) A common molecular basis for three inherited kidney stone diseases. Nature 379:445–449. https://doi.org/10.1038/379445a0

Ludwig M, Doroszewicz J, Seyberth HW, et al (2005) Functional evaluation of Dent’s disease-causing mutations: implications for ClC-5 channel trafficking and internalization. Hum Genet 117:228–237. https://doi.org/10.1007/s00439-005-1303-2

Mansour-Hendili L, Blanchard A, Le Pottier N, et al (2015) Mutation Update of the CLCN5 Gene Responsible for Dent Disease 1. Hum Mutat 36:743–752. https://doi.org/10.1002/humu.22804

Matsumoto A, Matsui I, Mori T, et al (2018) Severe Osteomalacia with Dent Disease Caused by a Novel Intronic Mutation of the CLCN5 gene. Intern Med 57:3603–3610. https://doi.org/10.2169/internalmedicine.1272-18

Matsuyama T, Awazu M, Oikawa T, et al (2004) Molecular and clinical studies of Dent’s disease in Japan: biochemical examination and renal ultrasonography do not predict carrier state. Clin Nephrol 61:231–237. https://doi.org/10.5414/cnp61231

Mo L, Xiong W, Qian T, et al (2004) Coexpression of complementary fragments of ClC-5 and restoration of chloride channel function in a Dent’s disease mutation. Am J Physiol, Cell Physiol 286:C79-89. https://doi.org/10.1152/ajpcell.00009.2003

Morimoto T, Uchida S, Sakamoto H, et al (1998) Mutations in CLCN5 chloride channel in Japanese patients with low molecular weight proteinuria. J Am Soc Nephrol 9:811–818

Nakazato H, Hattori S, Furuse A, et al (1997) Mutations in the CLCN5 gene in Japanese patients with familial idiopathic low-molecular-weight proteinuria. Kidney Int 52:895–900. https://doi.org/10.1038/ki.1997.410

Nakazato H, Yoshimuta J, Karashima S, et al (1999) Chloride channel CLCN5 mutations in Japanese children with familial idiopathic low molecular weight proteinuria. Kidney Int 55:63–70. https://doi.org/10.1046/j.1523-1755.1999.00231.x

Okamoto T, Tajima T, Hirayama T, Sasaki S (2012) A patient with Dent disease and features of Bartter syndrome caused by a novel mutation of CLCN5. Eur J Pediatr 171:401–404. https://doi.org/10.1007/s00431-011-1578-3

Picollo A, Pusch M (2005) Chloride/proton antiporter activity of mammalian CLC proteins ClC-4 and ClC-5. Nature 436:420–423. https://doi.org/10.1038/nature03720

Preston R, Naylor RW, Stewart G, et al (2020) A role for OCRL in glomerular function and disease. Pediatr Nephrol 35:641–648. https://doi.org/10.1007/s00467-019-04317-4

Ramos-Trujillo E, Garcia-Nieto V, Gonzalez-Acosta H, et al (2007a) Molecular analysis of the CLCN5 gene in Dent’s disease: first mutation identified in a patient from South America. Clin Nephrol 68:367–372. https://doi.org/10.5414/cnp68367

Ramos-Trujillo E, González-Acosta H, Flores C, et al (2007b) A missense mutation in the chloride/proton ClC-5 antiporter gene results in increased expression of an alternative mRNA form that lacks exons 10 and 11. Identification of seven new CLCN5 mutations in patients with Dent’s disease. J Hum Genet 52:255–261. https://doi.org/10.1007/s10038-007-0112-y

Saida K, Kamijo Y, Matsuoka D, et al (2014) A case of adult Dent disease in Japan with advanced chronic kidney disease. CEN Case Rep 3:132–138. https://doi.org/10.1007/s13730-013-0102-1

Sakakibara N, Nagano C, Ishiko S, et al (2020) Comparison of clinical and genetic characteristics between Dent disease 1 and Dent disease 2. Pediatr Nephrol. https://doi.org/10.1007/s00467-020-04701-5

Satoh N, Yamada H, Yamazaki O, et al (2016) A pure chloride channel mutant of CLC-5 causes Dent’s disease via insufficient V-ATPase activation. Pflugers Arch 468:1183–1196. https://doi.org/10.1007/s00424-016-1808-7

Schmieder S, Bogliolo S, Ehrenfeld J (2007) N-glycosylation of the Xenopus laevis ClC-5 protein plays a role in cell surface expression, affecting transport activity at the plasma membrane. J Cell Physiol 210:479–488. https://doi.org/10.1002/jcp.20882

Sekine T, Komoda F, Miura K, et al (2014) Japanese Dent disease has a wider clinical spectrum than Dent disease in Europe/USA: genetic and clinical studies of 86 unrelated patients with low-molecular-weight proteinuria. Nephrol Dial Transplant 29:376–384. https://doi.org/10.1093/ndt/gft394

Sekine T, Nozu K, Iyengar R, et al (2007) OCRL1 mutations in patients with Dent disease phenotype in Japan. Pediatr Nephrol 22:975–980. https://doi.org/10.1007/s00467-007-0454-x

Sethi SK, Ludwig M, Kabra M, et al (2009) Vitamin A responsive night blindness in Dent’s disease. Pediatr Nephrol 24:1765–1770. https://doi.org/10.1007/s00467-009-1198-6

Sheffer-Babila S, Chandra M, Speiser PW (2008) Growth hormone improves growth rate and preserves renal function in Dent disease. J Pediatr Endocrinol Metab 21:279–286. https://doi.org/10.1515/jpem.2008.21.3.279

Shrimpton AE, Hoopes RR, Knohl SJ, et al (2009) OCRL1 mutations in Dent 2 patients suggest a mechanism for phenotypic variability. Nephron Physiol 112:p27-36. https://doi.org/10.1159/000213506

Smith AJ, Lippiat JD (2010) Direct endosomal acidification by the outwardly rectifying CLC-5 Cl(-)/H(+) exchanger. J Physiol (Lond) 588:2033–2045. https://doi.org/10.1113/jphysiol.2010.188540

Smith AJ, Reed AAC, Loh NY, et al (2009) Characterization of Dent’s disease mutations of CLC-5 reveals a correlation between functional and cell biological consequences and protein structure. Am J Physiol Renal Physiol 296:F390-397. https://doi.org/10.1152/ajprenal.90526.2008

Solanki AK, Arif E, Morinelli T, et al (2018) A Novel CLCN5 Mutation Associated With Focal Segmental Glomerulosclerosis and Podocyte Injury. Kidney Int Rep 3:1443–1453. https://doi.org/10.1016/j.ekir.2018.06.003

Takemura T, Hino S, Ikeda M, et al (2001) Identification of two novel mutations in the CLCN5 gene in Japanese patients with familial idiopathic low molecular weight proteinuria (Japanese Dent’s disease). Am J Kidney Dis 37:138–143. https://doi.org/10.1016/s0272-6386(01)80067-6

Tang X, Brown MR, Cogal AG, et al (2016) Functional and transport analyses of CLCN5 genetic changes identified in Dent disease patients. Physiol Rep 4:e12776. https://doi.org/10.14814/phy2.12776

Tanuma A, Sato H, Takeda T, et al (2007) Functional characterization of a novel missense CLCN5 mutation causing alterations in proximal tubular endocytic machinery in Dent’s disease. Nephron Physiol 107:p87-97. https://doi.org/10.1159/000111253

Tasic V, Lozanovski VJ, Korneti P, et al (2011) Clinical and laboratory features of Macedonian children with OCRL mutations. Pediatr Nephrol 26:557–562. https://doi.org/10.1007/s00467-010-1758-9

Tosetto E, Ghiggeri GM, Emma F, et al (2006) Phenotypic and genetic heterogeneity in Dent’s disease--the results of an Italian collaborative study. Nephrol Dial Transplant 21:2452–2463. https://doi.org/10.1093/ndt/gfl274

Utsch B, Bökenkamp A, Benz MR, et al (2006) Novel OCRL1 mutations in patients with the phenotype of Dent disease. Am J Kidney Dis 48:942.e1–14. https://doi.org/10.1053/j.ajkd.2006.08.018

Vaisbich MH, Henriques LDS, Igarashi T, et al (2012) The long-term use of enalapril and hydrochlorothiazide in two novel mutations patients with Dent’s disease type 1. J Bras Nefrol 34:78–81

Valina MR, Larsen CP, Kanosky S, et al (2013) A novel CLCN5 mutation in a boy with asymptomatic proteinuria and focal global glomerulosclerosis. Clin Nephrol 80:377–384. https://doi.org/10.5414/CN107429

Wang X, Anglani F, Beara-Lasic L, et al (2016) Glomerular Pathology in Dent Disease and Its Association with Kidney Function. Clin J Am Soc Nephrol 11:2168–2176. https://doi.org/10.2215/CJN.03710416

Wang Y, Cai H, Cebotaru L, et al (2005) ClC-5: role in endocytosis in the proximal tubule. Am J Physiol Renal Physiol 289:F850-862. https://doi.org/10.1152/ajprenal.00011.2005

Wen M, Shen T, Wang Y, et al (2018) Next-Generation Sequencing in Early Diagnosis of Dent Disease 1: Two Case Reports. Front Med (Lausanne) 5:347. https://doi.org/10.3389/fmed.2018.00347

Wong W, Poke G, Stack M, et al (2017) Phenotypic variability of Dent disease in a large New Zealand kindred. Pediatr Nephrol 32:365–369. https://doi.org/10.1007/s00467-016-3472-8

Wrong OM, Norden AG, Feest TG (1994) Dent’s disease; a familial proximal renal tubular syndrome with low-molecular-weight proteinuria, hypercalciuria, nephrocalcinosis, metabolic bone disease, progressive renal failure and a marked male predominance. QJM 87:473–493

Yamamoto K, Cox JP, Friedrich T, et al (2000) Characterization of renal chloride channel (CLCN5) mutations in Dent’s disease. J Am Soc Nephrol 11:1460–1468

Yanagida H, Ikeoka M, Kuwajima H, et al (2004) A boy with Japanese Dent’s disease exhibiting abnormal calcium metabolism and osseous disorder of the spine: defective megalin expression at the brushborder of renal proximal tubules. Clin Nephrol 62:306–312. https://doi.org/10.5414/cnp62306

Ye Q, Shen Q, Rao J, et al (2020) Multicenter study of the clinical features and mutation gene spectrum of Chinese children with Dent disease. Clin Genet 97:407–417. https://doi.org/10.1111/cge.13663

Zaniew M, Bökenkamp A, Kolbuc M, et al (2018) Long-term renal outcome in children with OCRL mutations: retrospective analysis of a large international cohort. Nephrol Dial Transplant 33:85–94. https://doi.org/10.1093/ndt/gfw350

Zaniew M, Mizerska-Wasiak M, Załuska-Leśniewska I, et al (2017) Dent disease in Poland: what we have learned so far? Int Urol Nephrol 49:2005–2017. https://doi.org/10.1007/s11255-017-1676-x

Zhang Y, Fang X, Xu H, Shen Q (2017) Genetic Analysis of Dent’s Disease and Functional Research of CLCN5 Mutations. DNA Cell Biol 36:1151–1158. https://doi.org/10.1089/dna.2017.3731
